# Longitudinal Analysis of the Human B Cell Response to Ebola Virus Infection

**DOI:** 10.1016/j.cell.2019.04.036

**Published:** 2019-05-30

**Authors:** Carl W. Davis, Katherine J.L. Jackson, Anita K. McElroy, Peter Halfmann, Jessica Huang, Chakravarthy Chennareddy, Ashley E. Piper, Yvonne Leung, César G. Albariño, Ian Crozier, Ali H. Ellebedy, John Sidney, Alessandro Sette, Tianwei Yu, Sandra C.A. Nielsen, Arthur J. Goff, Christina F. Spiropoulou, Erica Ollman Saphire, Guy Cavet, Yoshihiro Kawaoka, Aneesh K. Mehta, Pamela J. Glass, Scott D. Boyd, Rafi Ahmed

**Affiliations:** 1 Emory Vaccine Center and Department of Microbiology and Immunology, Emory University, Atlanta, GA, USA; 2 Department of Pathology, Stanford University, Stanford, CA, USA; 3 Immunology Division, Garvan Institute of Medical Research, Darlinghurst, NSW, Australia; 4 Viral Special Pathogens Branch, US Centers for Disease Control and Prevention, Atlanta, GA, USA; 5 Division of Pediatric Infectious Disease, Emory University, Atlanta, GA, USA; 6 Division of Pediatric Infectious Disease, University of Pittsburgh, Pittsburgh, PA, USA; 7 Department of Pathobiological Sciences, School of Veterinary Medicine, University of Wisconsin-Madison, WI, USA; 8 Virology Division, United States Army Medical Research Institute for Infectious Diseases, Fort Detrick, MD, USA; 9 Atreca, Redwood City, CA, USA; 10 Department of Immunology and Microbial Science, The Scripps Research Institute, La Jolla, CA, USA; 11 Integrated Research Facility at Fort Detrick, Clinical Monitoring Research Program Directorate, Frederick National Laboratory for Cancer Research sponsored by the National Cancer Institutes, Frederick, MD, USA; 12 Division of Immunobiology, Department of Pathology and Immunology Washington University School of Medicine, St. Louis, MO, USA; 13 Division of Vaccine Discovery, La Jolla Institute for Allergy and Immunology, La Jolla, CA, USA; 14 Department of Medicine, University of California San Diego, La Jolla, CA, USA; 15 Department of Biostatistics and Bioinformatics, Emory University, Atlanta, GA, USA; 16 La Jolla Institute for Immunology, La Jolla, CA, USA; 17 Division of Virology, Department of Microbiology and Immunology, International Research Center for Infectious Diseases, Institute of Medical Science, University of Tokyo, Tokyo, Japan; 18 Division of Infectious Diseases, School of Medicine, Emory University, Atlanta, GA, USA

**Keywords:** Ebola, B cell repertoire, public clonotype, antibody evolution, IgG subclass

## Abstract

Ebola virus (EBOV) remains a public health threat. We performed a longitudinal study of B cell responses to EBOV in four survivors of the 2014 West African outbreak. Infection induced lasting EBOV-specific immunoglobulin G (IgG) antibodies, but their subclass composition changed over time, with IgG1 persisting, IgG3 rapidly declining, and IgG4 appearing late. Striking changes occurred in the immunoglobulin repertoire, with massive recruitment of naive B cells that subsequently underwent hypermutation. We characterized a large panel of EBOV glycoprotein-specific monoclonal antibodies (mAbs). Only a small subset of mAbs that bound glycoprotein by ELISA recognized cell-surface glycoprotein. However, this subset contained all neutralizing mAbs. Several mAbs protected against EBOV disease in animals, including one mAb that targeted an epitope under evolutionary selection during the 2014 outbreak. Convergent antibody evolution was seen across multiple donors, particularly among VH3-13 neutralizing antibodies specific for the GP1 core. Our study provides a benchmark for assessing EBOV vaccine-induced immunity.

## Highlights

• Ebola virus infection causes massive recruitment of naive B cells
• Virus-specific antibodies continue to class-switch and mutate for months after acute infection
• Protective antibodies can be neutralizing or non-neutralizing and can appear early
• Convergent, protective antibody rearrangements are seen in multiple donors

## Graphical Abstract

**Figure:**
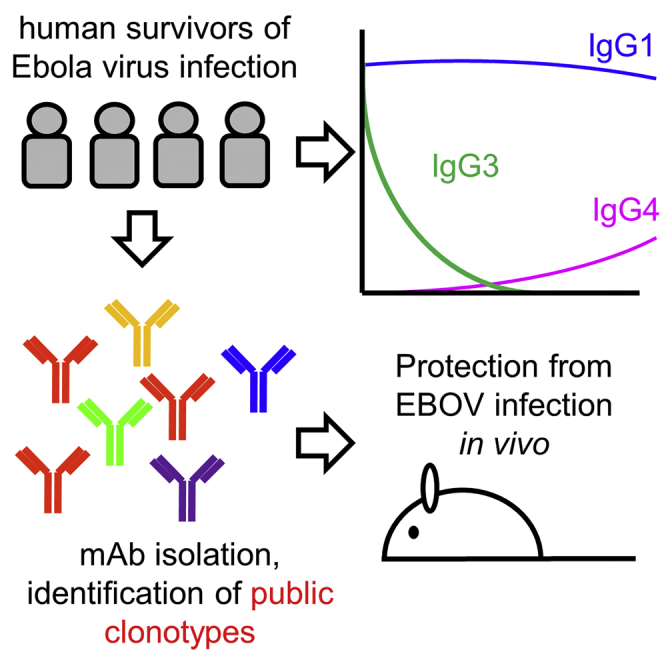

## Teaser

A longitudinal study of Ebola virus infection survivors maps out the antibody features that confer protection, with potential implications for vaccination.

## Introduction

The West African Ebola virus disease (EVD) outbreak of 2014–2016 was the largest ever recorded, responsible for more than 11,000 deaths (WHO, 2016). The experiences of the more than 17,000 survivors have reshaped our knowledge of human immune responses to Ebola virus (EBOV) infection. Although once thought to cause suppression of the host immune response (Geisbert and Jahrling, 2004), we now know that EBOV infection leads to extensive activation of B and T cells and high levels of inflammatory molecule expression during acute infection (McElroy et al., 2015, McElroy et al., 2016, Ellebedy et al., 2016, Ruibal et al., 2016). The robust B cell responses induced by EBOV infection eventually lead to the generation of highly potent protective antibodies, as shown by the ability of monoclonal antibodies (mAbs) isolated from human survivors to protect against EVD *in vivo* (Parren et al., 2002, Corti et al., 2016, Bornholdt et al., 2016, Flyak et al., 2016, Maruyama et al., 1999). However, these mAbs have typically been isolated several months or years after infection, so it is unclear what role they play in the initial control of infection.

Longitudinal serological studies in human survivors of EBOV infection, coupled with a detailed molecular profiling of their B cell responses, could help to answer whether protective antibodies appear early enough to help control acute infection. Such studies could also reveal whether there are common themes in the protective human immune response against EBOV that could be used to guide vaccine design and evaluation. Such studies are critically needed as EBOV continues to cause disease outbreaks, including the 2018 Équateur and Kivu outbreaks in the Democratic Republic of the Congo (WHO, 2018).

Between August and December of 2014, four EBOV-infected patients were treated at Emory University Hospital (Lyon et al., 2014, McElroy et al., 2015, Liddell et al., 2015, Kraft et al., 2015, Marshall Lyon et al., 2017, Varkey et al., 2015). All four patients agreed to enroll in a 3-year follow-up study, offering a unique opportunity to track the evolution of their immune responses. Here, we present an analysis of B cell responses in these four donors, focusing primarily on antibodies to the EBOV surface glycoprotein.

## Results

### Serum Antibody Responses in EVD Survivors

**Figure 1:**
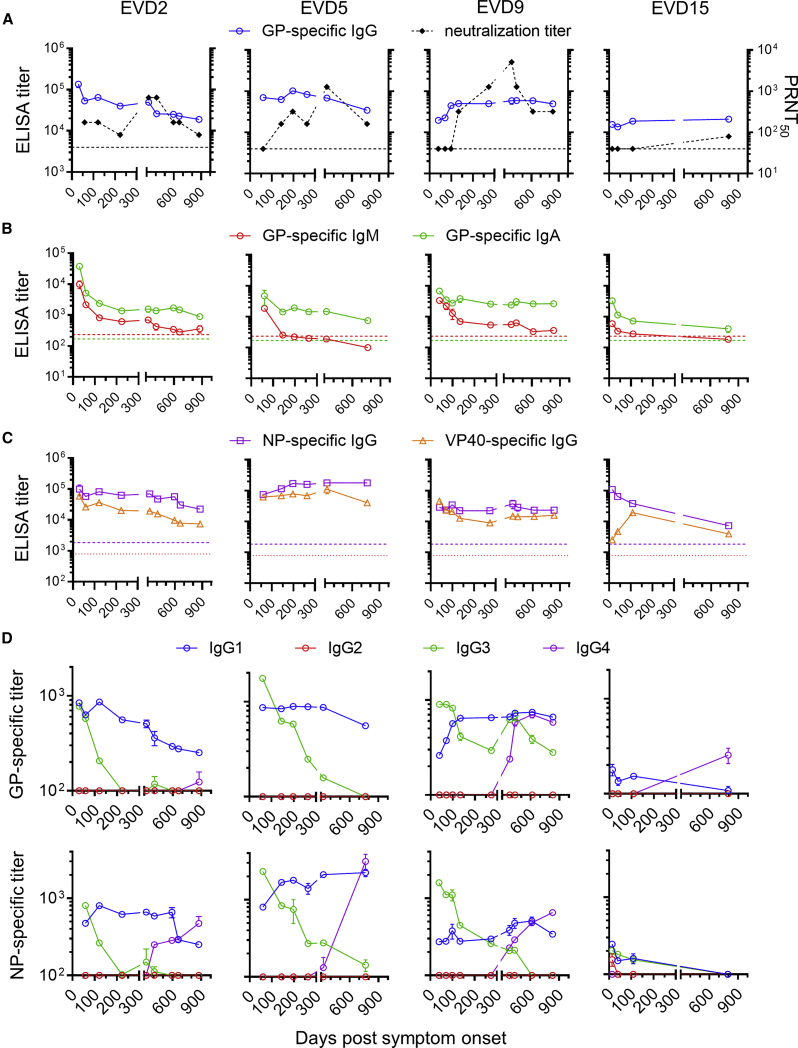
Antibody Responses in Ebola Virus Disease Survivors (A) Kinetics of GP-specific IgG and neutralizing titers. GP-specific plasma IgG was measured by ELISA. The average and standard deviation of two assays is shown. Titers in control donors were below the y axis limit. The 50% plaque reduction neutralization titer (PRNT_50_) shown is the average of two replicate assays. Dotted lines indicate PRNT assay detection limit. (B) GP-specific IgM and IgA responses. ELISA titers were determined using IgM or IgA detection reagents. Dotted lines indicate the mean titers of three control donors. (C) IgG responses to Ebola virus (EBOV) nucleoprotein (NP) and matrix protein (VP40). Dotted lines indicate the NP-specific ELISA titers of three control donors. VP40-specific titers were below the y axis limit. (D) Changes in EBOV-specific IgG subclasses over time. IgG1-, IgG2-, IgG3-, and IgG4-specific reagents were used to measure subclass-specific responses to GP (top) or NP (bottom). See also Figure S1.

Longitudinal blood samples were collected from the four EVD patients following their discharge from the hospital. Antibodies against EBOV glycoprotein (GP) were tracked by ELISA and virus-neutralizing antibodies were measured by plaque reduction assay (Figure 1A). At discharge, all four patients had high GP-specific immunoglobulin G (IgG) antibody titers that persisted over 2 years. GP-specific IgM fell over time to background levels, while IgA levels remained elevated (Figure 1B). All four donors had sustained IgG responses to EBOV nucleoprotein (NP) and matrix protein (VP40) (Figure 1C). In contrast to ELISA titers, neutralizing antibody responses were low or absent at discharge and rose slowly over 1 year (Figure 1A).

### Dynamic Changes in IgG Subclasses of EBOV-Specific Antibodies after Infection

**Figure S1:**
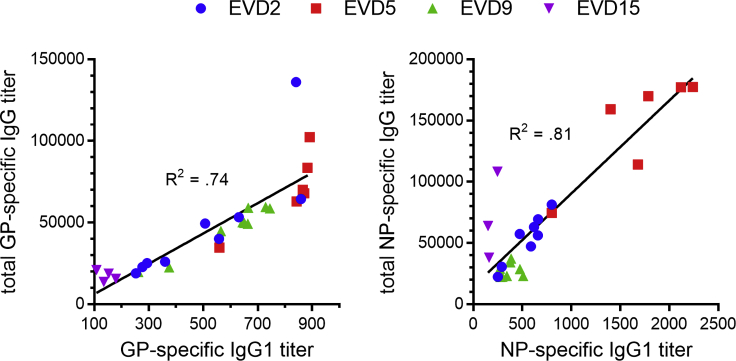
Correlation between EBOV-Specific IgG1 and Total EBOV-Specific IgG, Related to Figure 1 EBOV glycoprotein-specific titers are shown on the left and nucleoprotein-specific titers are shown on the right. The best fit linear regression lines and R^2^ values are shown.

We next examined the subclass composition of the GP-specific IgG response (Figure 1D, top). IgG1 levels were near peak at discharge and declined relatively slowly. GP-specific IgG1 correlated closely with total GP-specific IgG (Figure S1), suggesting that IgG1 made up most of the response. IgG2 was not detectable, while IgG3 was highest early and declined relatively rapidly, except in donor EVD9 where there was a second peak around 1 year after symptom onset. GP-specific IgG4 appeared in EVD2, EVD9, and EVD15 only at late time points (1–2 years post-infection). We observed similar patterns of subclass usage over time (sustained IgG1, declining IgG3, and delayed IgG4) when we examined NP-specific responses (Figure 1D, bottom).

### EBOV Infection Causes Global Changes in the Antibody Repertoire

**Figure 2:**
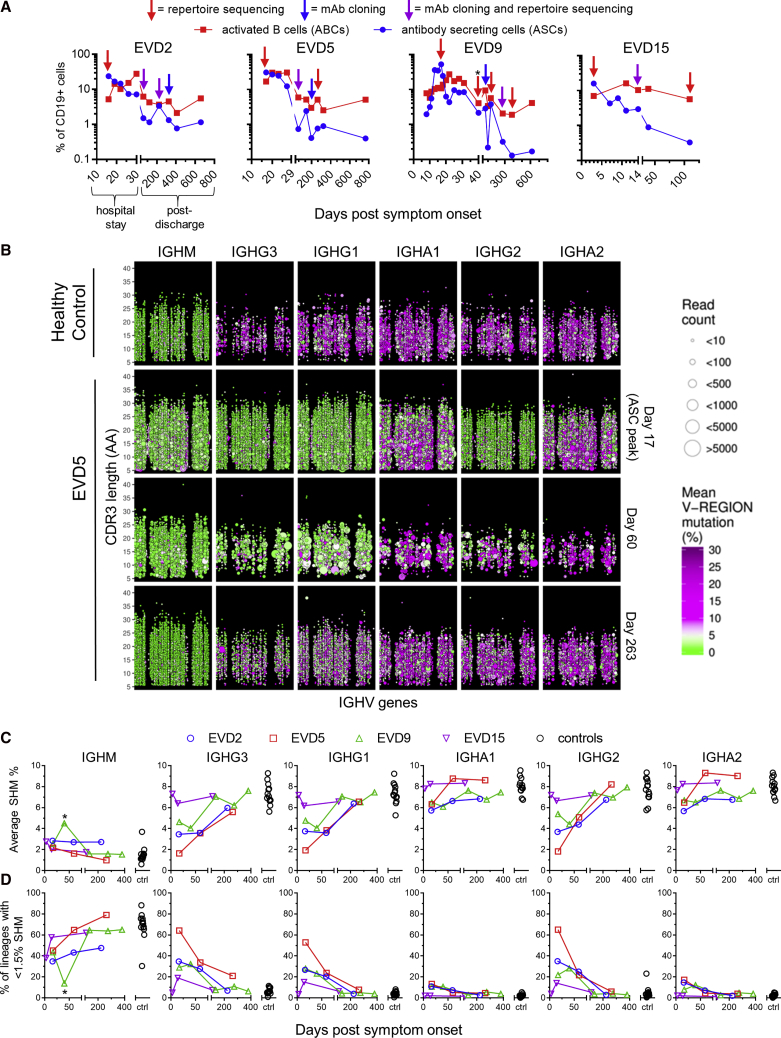
Profiling of Antibody Sequences in EVD Survivors (A) Frequencies of activated B cells (ABCs) and antibody secreting cells (ASCs). ABCs were defined as CD20^+^ CD19^+^ CD71^hi^ cells and ASCs as CD19^+^ CD38^hi^ CD27^hi^ CD20 cells. Arrows indicate samples used for repertoire sequencing or monoclonal antibody (mAb) production. The leftmost arrow on each plot corresponds to the ASC peak. Total PBMC RNA was used for repertoire sequencing except for EVD9 day 40 (^∗^), where RNA from magnetically sorted ASCs was used. (B) Overview of antibody repertoire in EVD5. The IGH repertoires of a control subject (row 1) and EVD5 (rows 2–4) are shown. Each point represents a single antibody lineage. Point sizes are proportional to the number of unique reads in each lineage, positions indicate the IGHV usage and CDR3 length, and colors indicate the mean somatic hypermutation (SHM). Points are jittered to prevent over-plotting of lineages with the same V and CDR3 lengths. Time points are in days post symptom onset. (C and D) Summary of somatic hypermutation levels in repertoire sequencing. The average SHM levels of all lineages (C) and the percentage of “low mutation” lineages with SHM <1.5% (D) are compared with 13 control subjects. ^∗^The second time point for EVD9 used sorted ASC RNA. See also Figure S2.

We performed longitudinal antibody repertoire sequencing to examine the global effects of EBOV infection on the B cell compartment. The first sample analyzed in each donor corresponded to the peak of the blood antibody secreting cell (ASC) response (Figure 2A). As we reported previously, 20%–60% of all B cells at this time point were ASCs (CD38hi/CD27hi) and another 5%–10% were activated B cells (ABCs; CD71^+^/CD20^+^) (McElroy et al., 2015, Ellebedy et al., 2016). A high frequency of antigen-specific cells are found among ASCs after vaccination or infection (Wrammert et al., 2008, Wrammert et al., 2012, Odendahl et al., 2005), and ABCs are likewise enriched for antigen-specific cells after vaccination (Ellebedy et al., 2016). We used peripheral blood mononuclear cell (PBMC) RNA for all repertoire sequencing except EVD9 day 40, where RNA from sorted ASCs was used.

**Figure S2:**
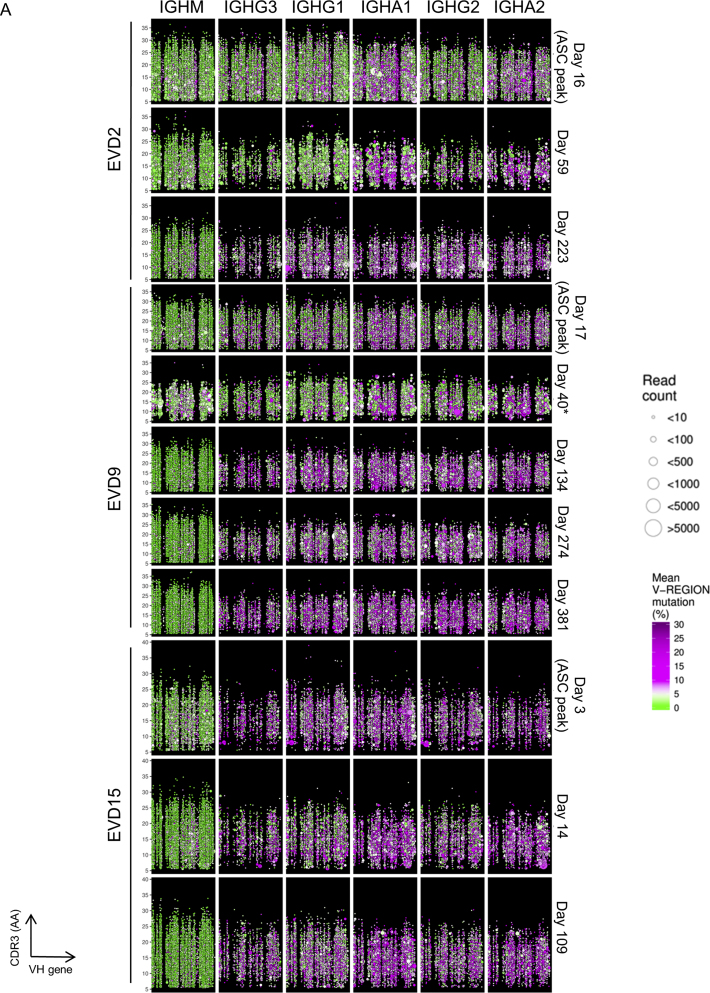
Overview of Antibody Repertoire at Various Time Points for EVD2, EVD9, and EVD15, Related to Figure 2 Each B cell lineage is represented as a point in the plot. The position of each point represents the clone’s isotype (column), time point (row), IGHV usage (x axis) and CDR3 length (y axis). Sizes indicate the number of unique reads grouped into the clone. Colors indicate the mean SHM% for the clone. Points are jittered to prevent some over-plotting of clones with the same V and CDR3 lengths. Time points indicate the number of days elapsed since symptom onset. Note, RNA for EVD9 day 40 (marked with an asterisk) was extracted from magnetically-sorted ASCs, while RNA for the other time points came from total PBMCs.

Repertoire sequencing of a representative healthy donor is shown in the top row of Figure 2B. IgM sequences have low somatic hypermutation (SHM; green circles) and show small clone sizes, while IgG sequences display significant SHM (purple circles) and evidence of clonal expansion (larger circle sizes). In contrast, sequences with low SHM dominated the IgG1, IgG2, and IgG3 compartments at the peak of the ASC response in EVD5 (Figure 2C, row 2). Low SHM was also seen in IgG sequences at this time point for subjects EVD2 and EVD9 (Figure S2) and in IgA sequences for all three donors (Figures 2B and S2). Thus, at the ASC peak, most IgG transcripts and a large fraction of IgA transcripts were made by cells recently recruited from the naive B cell pool. At the next time point analyzed, an increased prevalence of low mutation clones was still apparent, particularly among IgG1 and IgG3 sequences, the isotypes most commonly utilized by virus-specific antibodies (Ferrante et al., 1990). We note that the RNA template quantity from the EVD5 day 60 sample was limited, resulting in fewer total clones being detected in this sample (Figure 2B, third row).

The average SHM% seen in IGH sequences from the four EVD donors and 13 healthy controls is shown in Figure 2C. Figure 2D shows the percentage of lineages with low levels of SHM (<1.5% SHM). SHM levels in class-switched IGH transcripts from EVD2, EVD5, and EVD9 were reduced and the frequency of low SHM lineages increased relative to controls for 2–3 months. These changes were not seen in EVD15, who had the lowest viremia (McElroy et al., 2015).

### Evidence for Prolonged EBOV-Specific B Cell Activation

**Figure S3:**
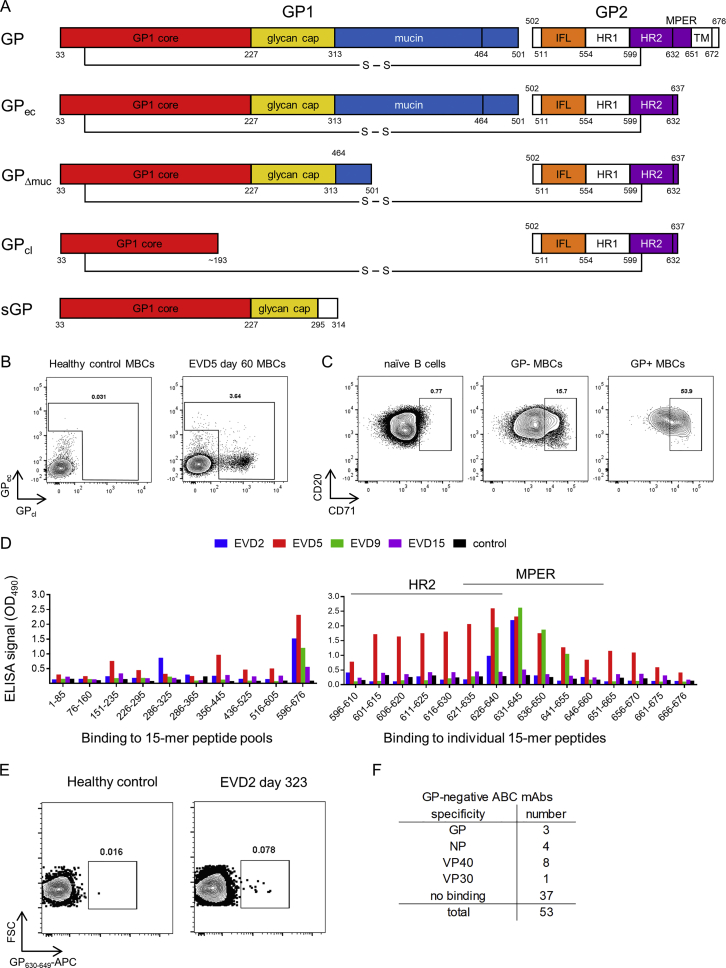
Strategies for Isolating GP-Specific mAbs, Related to Figure 3 A) Recombinant glycoprotein (GP) isoforms used for B cell sorting and antibody screening. The constructs shown are: GP = full length mature EBOV glyocoprotein (expressed on Jurkat cells and used for mAb screening); GP_ec_ = GP ectodomain residues 1-636 (used as sort probe and for ELISA); GP_Δmuc_ = GP delta mucin (used for ELISA); GP_cl_ = protease-cleaved GP (used as sort probe and for ELISA); sGP = secreted glycoprotein (used for ELISA). Legend: GP1 and GP2 = two GP subunits generated by furin cleavage at residue 501. S-S = disulfide bond linking GP1 and GP2. IFL = internal fusion loop. HR1 and HR2 = heptad repeat regions 1 and 2. MPER = membrane proximal extracellular region.TM = transmembrane domain. Region coloring matches that used for the antibody competition groups defined in this study. B) Gating strategy for identifying and sorting GP-specific B cells. PBMCs were stained with lineage markers and fluorescently labeled EBOV GP proteins and analyzed by flow cytometry. Example flow cytometry staining is shown for CD20+ IgD memory B cells (MBCs) from a healthy, EBOV-naive donor (left) or from patient EVD5 60 days after onset of viral symptoms (right). C) Expression of the activation marker CD71 by GP-binding cells. PBMCs from EVD5 day 60 are shown. Plots are gated on CD20+ IgD+ CD27 naive B cells (left), on MBCs that did not bind GP_ec_ or GP_cl_ (middle), or on MBCs that bound GP_ec_ and/or GP_cl_ (right). D) Detection of plasma antibody responses to short peptide epitopes from EBOV GP. Left: Diluted plasma from the four patients or a control donor was added to ELISA plates coated with pools of overlapping 15-mer peptides spanning EBOV GP, followed by detection of bound human IgG. Right: Plates were coated with the indicated 15-mer peptides from the C-terminal peptide pool (encompassing residues 596-676 of GP). ELISA results are shown for EVD2 and EVD9. E) Sorting MPER-specific B cells. A synthetic peptide spanning GP residues 630-649 was conjugated to APC and used to identify MPER-specific B cells from EVD2. F) Binding of ABC-derived mAbs to EBOV antigens. mAbs were tested for binding to ELISA wells coated with recombinant EBOV proteins. mAbs were considered GP-specific if they bound GP_ec_, GP_cl_, or GP_Δmuc_.

To track EBOV-specific B cells by flow cytometry, we used fluorescently labeled EBOV GP probes. We tested several GP isoforms as sort probes (Figure S3A) (Fusco et al., 2015). Two were selected for use: full-length GP ectodomain (GP_ec_) and cleaved GP (GP_cl_). The mucin and glycan cap domains have been removed from GP_cl_, exposing the NPC1 binding site (Chandran et al., 2005, Côté et al., 2011, Carette et al., 2011). A higher number of memory B cells bound GP_cl_ compared to GP_ec_ (Figure S3B), suggesting that the B cell response preferentially targeted epitopes that are exposed in GP_cl_ but masked in GP_ec_.

**Figure 3:**
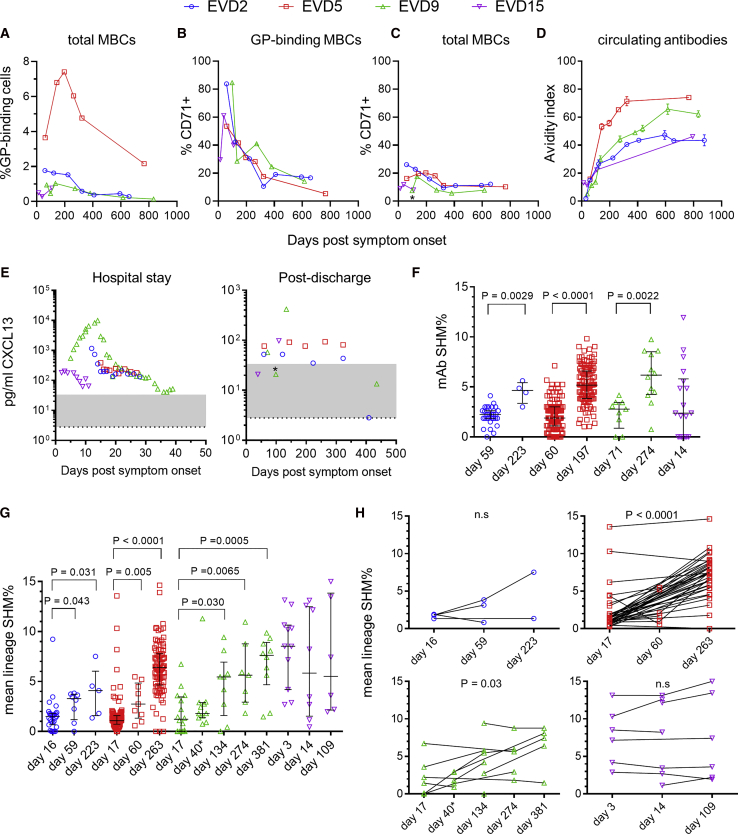
Evidence for Prolonged B Cell Activation following EBOV Infection (A) Frequency of GP-specific memory B cells over time. The percentage GP_cl_ binding cells is shown. (B and C) Prolonged activation of EBOV-specific MBCs. The percentage of cells expressing the activation marker CD71 is shown for GP_cl_ binding MBCs (B) or total MBCs (C). (D) Affinity maturation of GP-specific IgG. The avidity index is the percentage of GP-specific antibody (measured by ELISA) able to remain bound after washing with 8 M urea. The mean and standard deviation of three assays is shown. (E) Longitudinal CXCL13 levels in serum. Plasma levels of the germinal center-associated chemokine CXCL13 were determined using a commercial assay. The normal reference range is shaded gray and the limit of detection is indicated by the dotted line. ^∗^Sample taken shortly after uveitis episode. (F) Increase in SHM over time in GP-specific mAbs. Bars show the median SHM% and interquartile ranges. The p value for the Wilcoxon rank-sum test is shown. (G and H) SHM% of GP-binding IGH lineages from repertoire sequencing. Unpaired analysis of all lineages that included sequences from GP-binding cells is shown in (G). Paired analysis of GP-binding lineages detectable at multiple time points is shown in (H). Bars show the median SHM% and interquartile ranges. p values are for the Wilcoxon rank-sum test (G) or the Wilcoxon signed-rank test (H). ^∗^EVD9 day 40 sequences were derived from sorted ASC RNA. See also Figure S3 and Table S1.

GP-specific MBCs were highest in EVD5 but detectable in all four donors (Figure 3A). The activation/proliferation marker CD71 was expressed by most GP-binding B cells early after infection (Figure S3C) and remained elevated for 6–12 months (Figures 3B and 3C), suggesting continued antigen-driven activation of GP-specific cells. Consistent with this, there was a progressive increase in GP-specific antibody avidity during convalescence (Figure 3D). We found further evidence of ongoing immune activation when we examined plasma levels of the chemokine CXCL13, a known biomarker for germinal center activity (Havenar-Daughton et al., 2016). All four patients had elevated CXCL13 while hospitalized, and persistent elevation was seen for ∼1 year in donors EVD2, EVD5, and EVD9 (Figure 3E). There was a transient drop in CXCL13 in EVD9 after this patient was treated for EBOV-associated uveitis and received corticosteroids, which suppress CXCL13 production (Meraouna et al., 2006, Varkey et al., 2015).

To characterize the evolution of the EBOV-specific antibody response in more detail, we cloned antibodies from single GP_cl_ and/or GP_ec_ binding B cells. We obtained 723 unique paired heavy and light chain sequences, 367 of which were produced as mAbs (Table S1). 314 mAbs (86%) bound at least one EBOV GP isoform by ELISA. SHM in these GP-specific mAbs was low at early time points but increased by 6 months post-discharge in EVD2, EVD5, and EVD9 (Figure 3F). Due to limited sample quantity, GP-binding mAbs could only be sorted from EVD15 on the day of hospital discharge (day 14). Interestingly, higher SHM levels were seen in these mAbs, despite their early time of isolation.

We next examined our repertoire sequencing dataset for additional members of the GP-binding antibody lineages. SHM increased over time in these lineages in EVD2, EVD5, and EVD9 (Figures 3G and 3H). GP-binding lineages from EVD15 had higher SHM at the ASC peak compared to the other three subjects (p < 0.0001 by the Wilcoxon rank-sum test) and did not show increased SHM over time.

### Isolation of mAbs from MPER-Specific B Cells and Activated B Cells

We utilized additional strategies for isolating EBOV-specific antibodies from B cells beyond GP_cl/ec_ staining. We screened plasma from the four survivors for IgG against a library of 15-mer peptides from GP, in case some of these peptides were not present or accessible on the GP sort probes. IgG from all four donors bound to peptides in heptad repeat region 2 (HR2) and the membrane proximal extracellular region (MPER) of GP (Figure S3D), perhaps commensurate with the linear presentation of these sequences in the GP structure. We used a fluorescently labeled MPER peptide to sort B cells for mAb cloning (Figure S3E) and isolated a single MPER-specific mAb from EVD2. As an additional strategy to capture EBOV-specific B cells, we sorted CD71^+^ ABC from early time points that failed to bind GP_ec/cl_. 53 mAbs were isolated from GP_ec_/GP_cl_ negative CD71^+^ ABCs from EVD5 1 month after discharge. Sixteen (30%) were specific for an EBOV antigen (Figure S3F), with VP40 the most common target. Three mAbs (5%) bound GP by ELISA and were evaluated along with the MPER mAb and the mAbs from GP_cl/ec_ binding cells.

### Neutralization and GP-Binding Properties of mAbs

**Figure 4:**
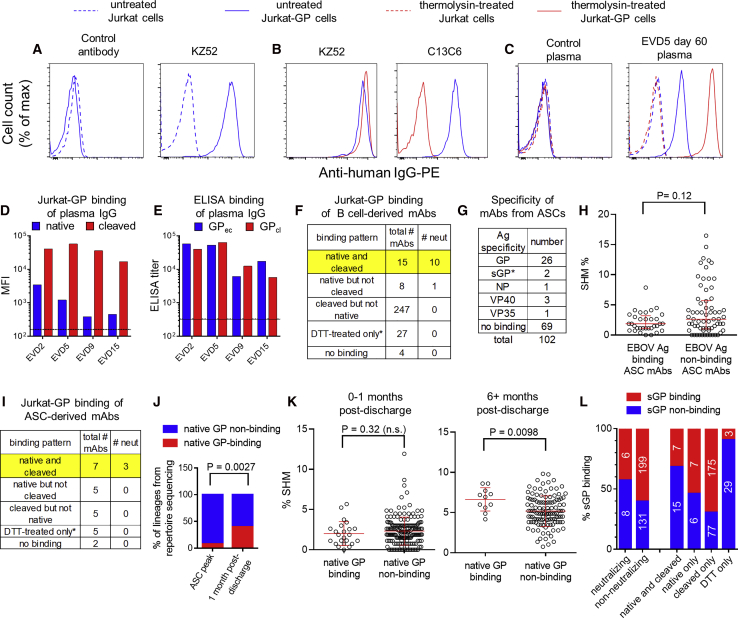
Binding Properties of GP-Specific mAbs (A–C) Cell surface binding assay. Histograms show the binding of mAbs (A and B) or plasma IgG (C) to Jurkat or Jurkat-GP cells. Thermolysin treatment was used to remove the glycan cap and mucin domains from GP. mAb KZ52 recognizes the GP base and mAb c13C6 recognizes the glycan cap. (D) Binding of plasma IgG to native versus cleaved GP on cells. Untreated (native) or thermolysin-treated (cleaved) Jurkat-GP cells were stained with Ebola virus disease (EVD) patient plasma as in (C). The mean fluorescence intensity (MFI) is shown. Lines indicate the mean binding of IgG from three control donors to untreated cells (solid line) or thermolysin-treated cells (dashed line). (E) Binding of plasma IgG to GP_ec_ versus GP_cl_ by ELISA. Endpoint IgG ELISA titers are shown. Average titers for three control donors are shown for GP_ec_ (solid line) and GP_cl_ (dashed line). (F) Summary of B cell-derived mAb binding to cells. Binding was assessed on untreated (native) or thermolysin-treated (cleaved) Jurkat-GP cells. ^∗^mAbs that failed to bind to native or cleaved GP were tested for binding to Jurkat-GP cells treated with DTT. (G) Specificity of mAbs from antibody secreting cells (ASCs). mAbs were cloned from EVD2 or EVD5 blood ASCs 1 month after discharge and their antigen specificities determined by ELISA. ^∗^mAbs were classified as sGP-specific if they bound sGP but not GP_ec_, GP_cl_, or GP_Δmuc_. (H) SHM levels in ASC-derived mAbs. The SHM% of mAbs that did or did not bind to an EBOV antigen are shown. Error bars indicate medians and interquartile ranges. The p value for the Wilcoxon rank-sum test is shown. (I) Summary ASC-derived mAb binding to cells. As in (F). (J) Increase in native GP-specific lineages 1 month after discharge. IGH lineages from the repertoire sequencing that included GP-specific mAbs were classified as native-GP binding if at least one of the included mAbs bound untreated Jurkat-GP cells. The p value shown is for Fisher’s exact test. (K) Increased accumulation of SHM in native GP-binding mAbs over time. The p value for the Wilcoxon rank-sum test is shown. (L) sGP binding of mAbs by category. mAbs were screened for sGP binding by ELISA and are classified according to their neutralization activity (left columns) or their Jurkat-GP binding phenotype (right columns). See also Figure S4 and Table S1.

**Figure S4:**
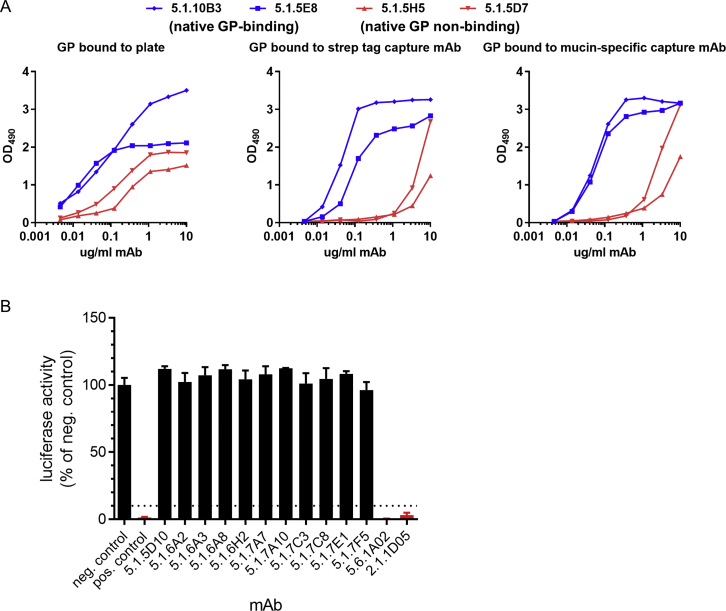
Additional Characterization of mAb Screening Assays, Related to Figure 4 A) Effects of GP antigen attachment strategy on ELISA results. ELISA plates were coated with GP_ec_ directly (left), or GP_ec_ was bound to plates coated with a capture antibody directed against the affinity purification tag on the C terminus of GP (StrepMAB-Classic, middle), or GP_ec_ was bound to plates coated with a mucin domain-specific capture mAb (h13F6; right). Plates were incubated with four biotinylated EBOV-specific mAbs and binding was detected with avidin-HRP. All four mAbs bound to GP_ec_ in the initial screening ELISA but mAbs 5.1.10B3 and 5.1.5E8 (blue) bound to native GP on Jurkat cells, whereas mAbs 5.1.5H5 and 5.1.5D7 did not. The two native GP-specific mAbs were relatively unaffected by the strategy used to attach GP_ec_ to the plate, while the two mAbs that do not bind native GP on Jurkat cells, showed reduced binding to GP_ec_ that was captured indirectly. B) Representative neutralization screening assay using biologically-contained EBOV. Luciferase-expressing EBOV lacking the VP30 gene was incubated with the indicated mAbs at 10 μg/ml for 2 hours at 37°C, then added to VP30-expressing Vero cell cultures in duplicate wells. Luciferase activity was measured three days later. mAbs were considered neutralizing if the luciferase signal was reduced by > 90% compared to the average signal in six wells incubated with virus plus negative control antibody. Bars for neutralizing mAbs are colored red. Neg. control = VP35-specific mAb 5-69.3.2. Pos. control = mAb 226/8.1. The SD of three replicate assays is shown.

To measure binding of mAbs to GP displayed in a similar orientation to that on viral particles, we established a stable GP-expressing cell line (Jurkat-GP). EBOV-specific mAbs selectively bound to Jurkat-GP cells and not the parental line (Figure 4A). We tested whether these cells could be treated with thermolysin to cleave off the glycan cap and mucin domains of GP, as previously demonstrated for pseudovirus particles (Kaletsky et al., 2007). As expected, thermolysin treatment prevented the binding of glycan cap-specific mAb c13C6 (Olinger et al., 2012, Wilson et al., 2000) but not mAb KZ52 (Maruyama et al., 1999) that binds to the GP base (Figure 4B). We then used these cells to assess binding of plasma IgG from the EVD survivors (Figures 4C and 4D). Interestingly, IgG binding for all four donors was 10- to 100-fold higher to thermolysin-cleaved GP than to native GP on Jurkat cells (Figure 4D). This matches our earlier findings that most GP-specific MBCs bound soluble GP_cl_ better than GP_ec_ by flow cytometry (Figure 3A). Surprisingly, selective binding of serum IgG to GP_cl_ was not apparent by ELISA (Figure 4E). This appeared to be due, at least in part, to changes in epitope accessibility when GP_ec_ was bound directly to the ELISA plate (Figure S4A), similar to previous findings (Hangartner et al., 2006).

We next used a biologically contained luciferase-expressing EBOV to screen our panel of mAbs for neutralization activity (Figure S4B) (Halfmann et al., 2008). Overall, only 11 mAbs out of 318 isolated from GP-binding B cells (∼3.5%) were neutralizing. Neutralization was strongly correlated with the ability of a given mAb to bind to native GP on Jurkat-GP cells (Figure 4F). The majority of the non-neutralizing mAbs bound epitopes that were exposed only after thermolysin treatment. Thirty-five mAbs failed to bind either native or cleaved GP on Jurkat cells despite binding GP by ELISA. Most of these mAbs were able to bind Jurkat-GP cells treated with the reducing agent DTT, which has been shown to increase exposure of epitopes on the GP base (Francica et al., 2010).

### Native GP-Specific Antibodies Are Enriched in ASCs Early during Convalescence

We next sought to characterize mAbs from ASCs. As we reported previously, EVD2 and EVD5 both showed a strong, ongoing blood ASC response 1 month after discharge (day 59/60 post symptom onset), much of which was EBOV-specific (McElroy et al., 2015). We sorted ASCs (without antigen labeling), cloned and expressed mAbs, and characterized their specificity (Figure 4G; Table S1). Approximately one third of the 102 ASC mAbs bound an EBOV antigen, with GP the most common target. The difference in the median hypermutation levels between EBOV antigen-binding versus non-binding mAbs was not statistically significant (Figure 4H). SHM levels in both groups of mAbs were lower than we have previously seen in ASCs derived from recall responses (Priyamvada et al., 2016, Li et al., 2012).

Interestingly, half of the GP-specific antibodies from ASCs (12 of 24) were able to target the native GP protein expressed on Jurkat cells, and 3 of these were neutralizing (Figure 4I). The percentage of ASCs expressing native GP-specific antibodies (50%) was significantly higher than what was seen for GP-specific memory B cells (7%); p = 0.006 by Fisher’s exact test. Thus, functionally relevant antigen specificities were enriched in ASCs early after resolution of acute infection. We examined whether this enrichment also could be seen in our repertoire sequencing from the peak of the ASC response during acute infection (Figure 4J). Interestingly, we found the opposite: at the ASC peak, only 7 out of 92 (7.6%) detectable GP-specific antibody lineages included native GP binding mAbs. In contrast, at 1 month post-discharge, 6 out of 15 detectable GP-specific lineages (40%) included native GP-binding mAbs. The difference in frequencies of native GP-binding lineages at the two time points was significant (p = 0.0027 by Fisher’s exact test). Thus, B cell epitopes that are not exposed on native GP were immunodominant during acute infection, while native GP was more commonly targeted during convalescence. Consistent with this, during the 6–10 months following acute infection, native GP-specific mAbs acquired more SHM, suggesting they underwent more rounds of selection in germinal centers during this period (Figure 4K).

It has been proposed that EBOV secreted glycoprotein (sGP) subverts the antibody response, increasing antibody responses toward sGP epitopes at the expense of responses to epitopes elsewhere on GP (Mohan et al., 2012). We therefore asked whether the high frequency of mAbs that were unable to bind to native GP or neutralize EBOV could be explained by sGP cross-reactivity. As seen in Figure 4L, ∼40% of neutralizing mAbs bound to sGP by ELISA versus ∼60% of non-neutralizing mAbs. Likewise, 40% of native GP-binding mAbs recognized sGP, versus 60% of native GP non-binding mAbs. However, even if sGP-binding mAbs were excluded from the analysis, only 17% (21 out of 127) of the remaining mAbs were able to bind native GP. Thus, sGP cross-reactivity alone cannot explain the immunodominance of GP epitopes that are not displayed on the native protein.

### Identification of Binding Sites for Neutralizing mAbs

**Figure 5:**
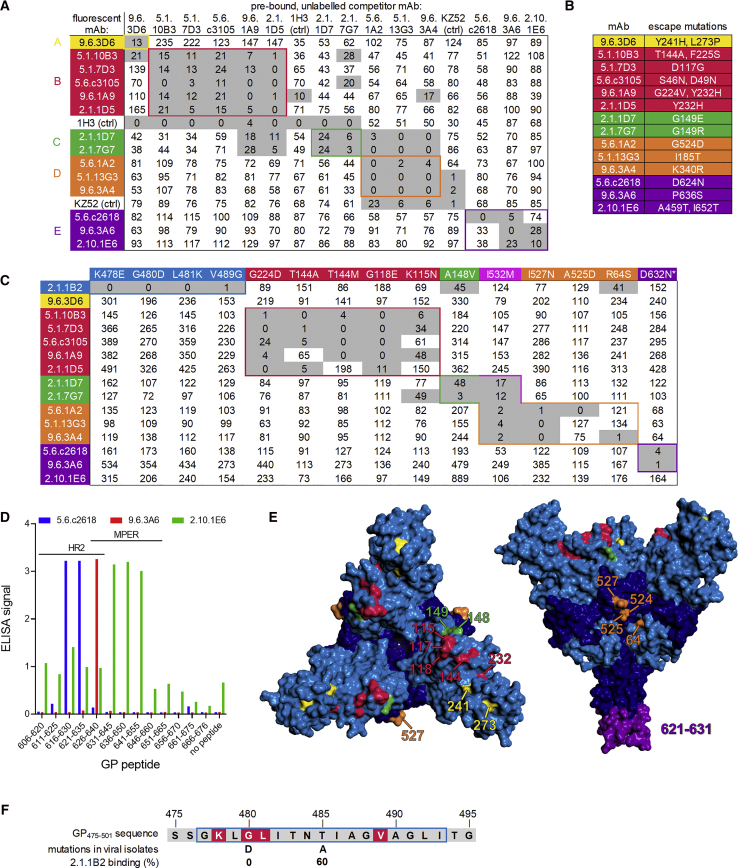
Determination of mAb Binding Sites (A) Competition binding of neutralizing mAbs. Jurkat-GP cells were pre-incubated with unlabeled competitor mAbs and stained with fluorescent mAbs. Numbers indicate specific fluorescence as a percent of the fluorescence without competitor. Gray shading indicates >70% reduction of binding. Five epitope groups (A through E) were defined. mAb names incorporate the donor number and the timing of the sample collection (e.g., 9.6.3D6: EVD9 6 months post-discharge sample, plate 3, well D6). (B) Summary of viral escape mutants. EBOV was grown in the presence of the indicated neutralizing mAbs and the GP genes of outgrown viruses were sequenced. (C) Binding of mAbs to GP mutant Jurkat cell lines. Binding to mutants is shown as a percentage of binding to wild type GP, after normalizing for GP expression levels. Gray shading indicates >50% decrease in binding. mAb 2.1.6F2 (mucin domain-specific) was used to normalize GP expression across cell lines. Similar results were obtained when KZ52 was used for normalization. ^∗^Mutation D632N creates a potential N-linked glycosylation site. (D) ELISA binding of group E mAbs to 15-mer peptides from the GP C terminus. (E) Mapping of binding sites. The EBOV glycoprotein trimer (PDB: 5JQ3) is shown. Left: top down view. Right: side view. Residues are colored by mAb competition group. (F) Effect of mutations on mAb 2.1.1B2 binding. GP residues 575–595 are shown. Locations of 2.1.1B2 binding mutations from (C) are in red. Blue box, mAb 14G7 epitope. Normalized 2.1.1B2 binding is shown for cells expressing the G480D and T485A mutants seen in West African isolates. See also Figure S5 and Table S1.

**Figure S5:**
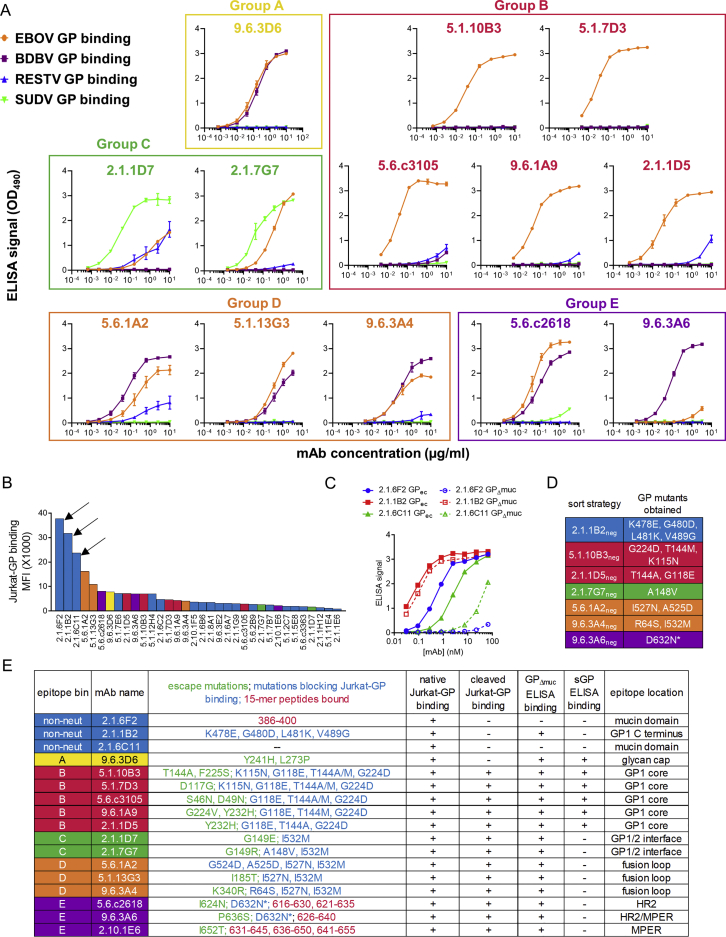
Characterization of mAb Specificity and Binding Sites, Related to Figure 5 A) Binding of neutralizing mAbs to glycoproteins from *Ebolavirus* species. Serial dilutions of each antibody were bound to plates coated with recombinant GP ectodomains from EBOV, Sudan virus (SUDV), Bundibugyo virus (BDBV), or Reston virus (RESTV). Bound antibodies were detected by ELISA. The OD is shown. B) Selection of non-neutralizing mAbs for further testing. The rank-ordered binding of mAbs to Jurkat-GP cells is shown. Unlabeled mAbs were incubated with Jurkat-GP cells at 5 μg/ml, washed, and incubated with fluorescently-labeled anti-human IgG. The mean fluorescence intensity (MFI) was measured by flow cytometry. For neutralizing mAbs, bars are colored by epitope group as in Figure 4. Non-neutralizing mAbs are shown as blue bars. mAbs 2.1.6F2, 2.1.1B2, and 2.1.6C11 (arrows) were selected for epitope mapping and testing in mice based on their high levels of binding in this assay. C) Non-neutralizing antibodies 2.1.6F2 and 2.1.6C11 require the mucin domain for binding. ELISA wells were coated either with full-length GP ectodomain (GP_ec_) or to GP lacking mucin domain residues 312-462 (GP_Δmuc_). Binding was assessed by ELISA. D) GP mutations identified in Jurkat-GP cell lines. Cells were transduced at low MOI with a lentiviral library containing randomly mutated EBOV GP. Cells which showed minimal binding to the indicated mAbs but normal binding to a panel of control mAbs were sorted and expanded as cell lines. GP mutations were identified by sequencing. Cell lines whose GP sequences contained more than one non-silent mutation were excluded from analysis. ^∗^the D632N mutation adds a potential N-linked glycosylation site. E) Summary of mapping studies for all mAbs.

We identified 14 strongly neutralizing mAbs in total and classified them into 5 groups (groups A–E) based on competitive binding to Jurkat-GP cells (Figure 5A). Groups A, B, and C all blocked the binding of glycan cap-specific antibody 1H3 (Qiu et al., 2011), suggesting they bound near the top of GP, while antibodies in group D competed with KZ52 (Maruyama et al., 1999), a neutralizing antibody specific for the GP chalice base (Lee et al., 2008). Group E antibodies competed with neither 1H3 nor KZ52. Each group displayed distinct preferences for binding to recombinant GP from different *Ebolavirus* species (Figure S5A).

We mapped the binding sites of these 14 mAbs as well as three non-neutralizing mAbs (2.1.6F2, 2.1.1B2, and 2.1.6C11) that showed unusually strong binding to Jurkat-GP cells (Figure S5B). We first evaluated mAb binding to different GP isoforms. mAbs 2.1.6F2 and 2.1.6C11 were able to bind GP_ec_ but not mucin-deleted GP (GP_Δmuc_), suggesting their epitopes are in the mucin domain (Figure S5C). mAbs 2.1.1B2 and 9.6.3D6 were able to bind to native but not cleaved GP on Jurkat cells and were able to bind GP_Δmuc_ by ELISA (Table S1), suggesting they bound either the glycan cap or the GP1 C terminus, which are present on GP_Δmuc_ but absent on cleaved GP.

We next grew EBOV in the presence of each neutralizing mAb and sequenced the GP genes of escape mutant viruses (Figure 5B). As an additional strategy that would also work for non-neutralizing antibodies, we used cell surface display to identify GP mutations that blocked mAb binding. We expressed a library of random EBOV GP mutants on Jurkat cells, sorted cell lines that were deficient for binding specific mAbs, and identified the GP mutations in these lines (Figures 5C and S5D). Finally, we screened all mAbs for binding to the library of overlapping 15-mer GP peptides. All three antibodies in group E bound to peptides from the HR2 and MPER regions (Figure 5D).

The results of the mapping studies are summarized in Figure S5E. Residues involved in binding of neutralizing mAbs are highlighted on the EBOV GP crystal structure (Figure 5E) (Lee et al., 2008, Zhao et al., 2016). Escape mutations for the single group A mAb (9.6.3D6) mapped to the glycan cap. Antibodies in competition group B appear to interact with residues on the GP1 core, the same region bound by mAb114, a protective human antibody isolated from a survivor of the 1995 Kikwit EBOV outbreak (Corti et al., 2016, Misasi et al., 2016). Group C mAbs were affected by mutations near the GP1-GP2 interface, while group D antibodies were affected by mutations in the GP fusion loop.

Amino acids involved in mAb 2.1.1B2 binding were located near the C terminus of GP1, in the same region recognized by protective mouse antibodies 12B5 and 14G7 (Wilson et al., 2000, Olal et al., 2012). This region appeared to be under selection during the 2014–2016 West African EBOV epidemic; mutations at positions 479 (Ladner et al., 2015), 480 (Urbanowicz et al., 2016, Ladner et al., 2015, Simon-Loriere et al., 2015), 484 (Pontremoli et al., 2016), or 485 (Urbanowicz et al., 2016, Park et al., 2015) were seen in viral isolates from the outbreak. One such mutations, G480D, arose multiple times in West Africa and was seen in 2% of viral isolates in one study (Urbanowicz et al., 2016, Ladner et al., 2015). This mutation was identified using our random mutagenesis approach and completely blocked 2.1.1B2 binding. Another mutation found in West African EBOV isolates, T485A, was of interest since it eliminates binding of the mouse antibody 14G7 to GP (Olal et al., 2012). We introduced the T485A change into GP by site-directed mutagenesis and found it decreased 2.1.1B2 binding by only 40% (Figure 5F). Thus, the mechanism of 2.1.1B2 binding to GP is similar but distinct from that of 14G7.

### Assessing mAb Neutralization Potency and Protective Efficacy

**Figure 6:**
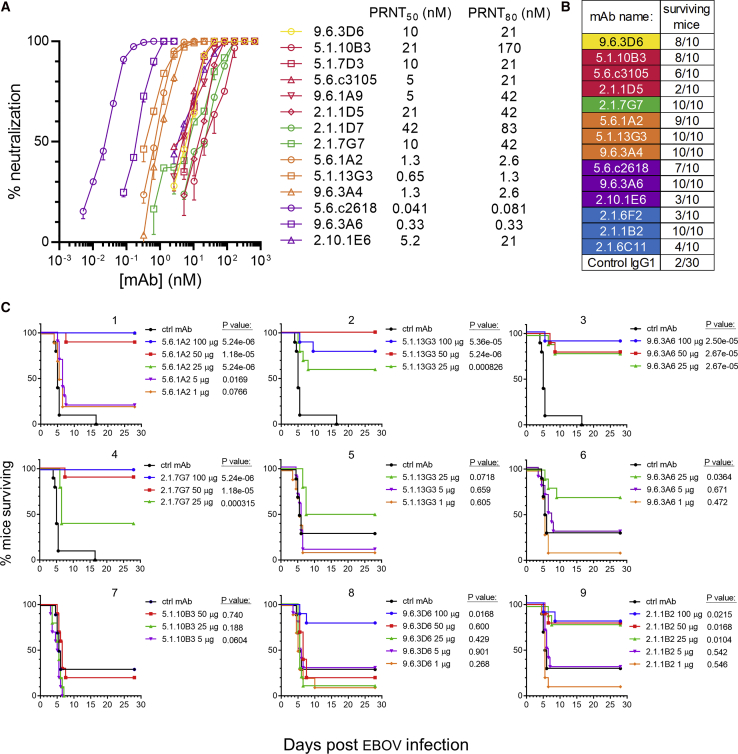
Assessment of mAb Neutralization and Mouse Protection (A) Quantification of mAb neutralization. PRNT assays were performed on Vero cells. The SD of two replicate assays is shown. (B) mAb-mediated protection of mice. 100 μg of each mAb was given 24 h prior to EBOV infection. Mice were followed for 28 days to assess survival. (C) Dose-down mouse protection studies. Selected mAbs were tested for their ability to protect mice at lower doses (graphs 1–4: experiment 1, graphs 5–9: experiment 2). Kaplan-Meier survival curves are shown. Curves are nudged slightly to prevent overlap. The p value for the log-rank test is shown. See also Figure S6 and Table S1.

**Figure S6:**
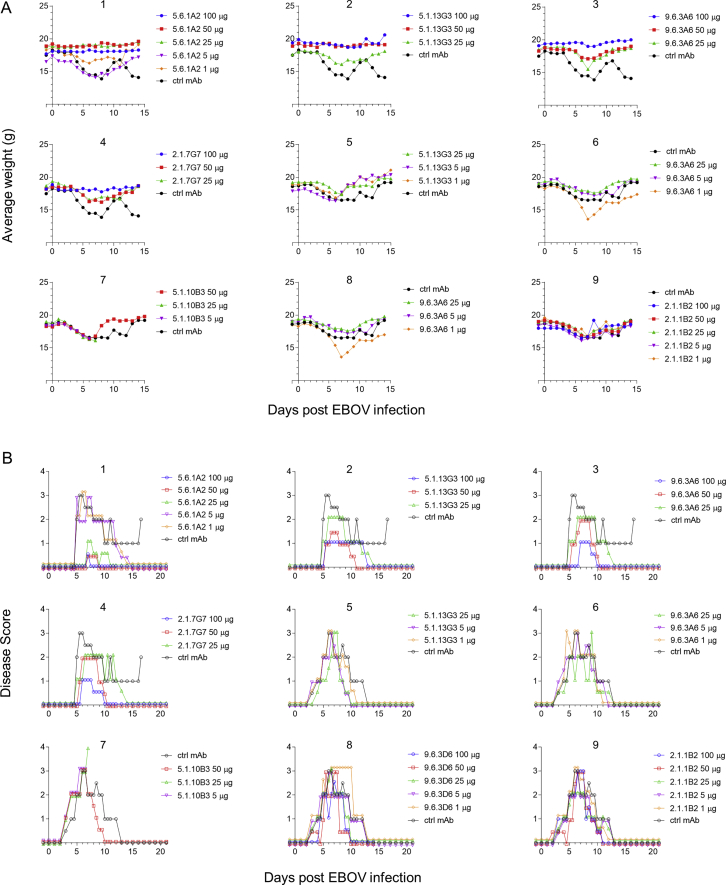
Weights and Disease Scores of Mice in EBOV Challenge Experiments, Related to Figure 6 A) Mouse weights. Mice were given the indicated antibody doses one day prior to infection with EBOV. The average daily weights of surviving mice in each treatment group is shown. Curves are nudged slightly to prevent overlap. Two separate infection experiments were performed (graphs 1-4: experiment 1, graphs 5-9: experiment 2). B) Disease scores. Mice in each group were assessed daily and assigned a disease score from 0 to 4 based on increasing severity of symptoms (see STAR Methods).

We next measured the neutralization potency of our panel of mAbs as well as their ability to protect against lethal EBOV challenge in mice. Antibodies recognizing the fusion loop and the HR2 regions of GP were the strongest neutralizers (Figure 6A). The mAbs provided varying degrees of protection to mice against lethal EBOV challenge when administered 24 h prior to infection (Figure 6B). For seven antibodies that were strongly protective at the initial dose tested (100 μg), we performed dose-down testing (Figure 6C). Weight loss and clinical disease scores in these experiments are shown in Figures S6A and S6B.

Four strongly protective mAbs were isolated from the donors as early as 2 months after symptom onset. These included non-neutralizing mAb 2.1.1B2 and neutralizing mAbs 2.1.7G7, 5.1.10B3, and 5.1.13G3. IGH sequences belonging to the 2.1.1B2 lineage could be detected in our repertoire sequencing at the peak of the ASC response in EVD2 (0.0078% of lineages at the ASC peak, increasing to 0.022% of lineages at day 59). At this time, EBOV RNA was still detectable in the blood (McElroy et al., 2015), meaning that 2.1.1B2-like antibodies were present early enough to potentially play a role during acute infection. Two other antibodies appeared later in the repertoire sequencing: 2.1.7G7 was detectable at day 59 (0.066% of reads in EVD2) and 5.1.10B3 was detected at both day 60 (0.41% of lineages in EVD5) and day 263 (0.00057% or lineages). 5.1.13G3 was not detectable in the repertoire sequencing at any time point.

### Detection of Convergent Antibody Rearrangements

**Figure S7:**
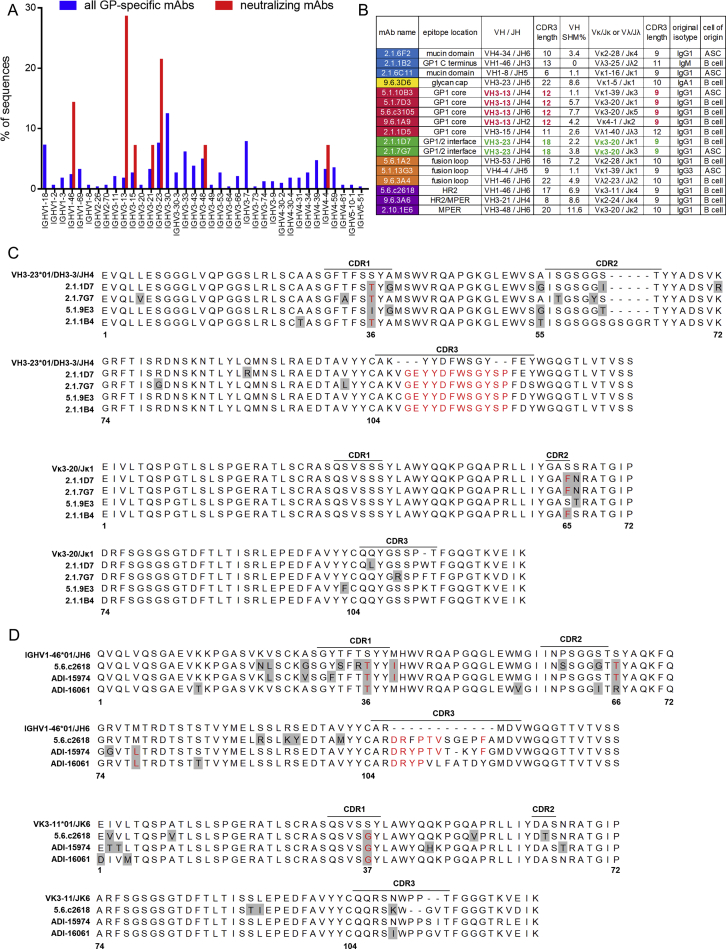
Sequence Features of Convergent mAbs, Related to Figure 7 A) Summary of V region usage by GP-specific mAbs identified in this study. V region usage was determined using IMGT/V-Quest. B) Overview of neutralizing mAb sequence features. V-genes and CDR3 lengths are highlighted in bold for mAbs within an epitope group that share common features. “Original isotype” refers to the heavy chain constant region expressed by the cell of origin for each antibody as determined by the initial sequencing PCR. (All VH genes were cloned into a human IgG1 expression vector for mAb production). C) Alignment of group C neutralizing mAbs 2.1.1D7 and 2.1.7G7 with non-neutralizing mAbs 5.1.9E3 and 2.1.1B4. Top: Heavy chain alignments. Bottom: Light chain alignments. Residues that differ from the germline V region are highlighted in gray. Red text indicates residues that are either mutated from the germline or derived from the germline DH region and shared among multiple EBOV-specific antibodies. IMGT codon numbering is shown below the sequences. D) Alignment of HR2-specific antibodies utilizing the VH1-46 heavy chain gene and VK3-11 light chain gene identified in this study and in Bornholdt et al. (2016). Top: heavy chain alignments. Bottom: light chain alignments. IMGT codon numbering is shown below the sequences.

**Figure 7:**
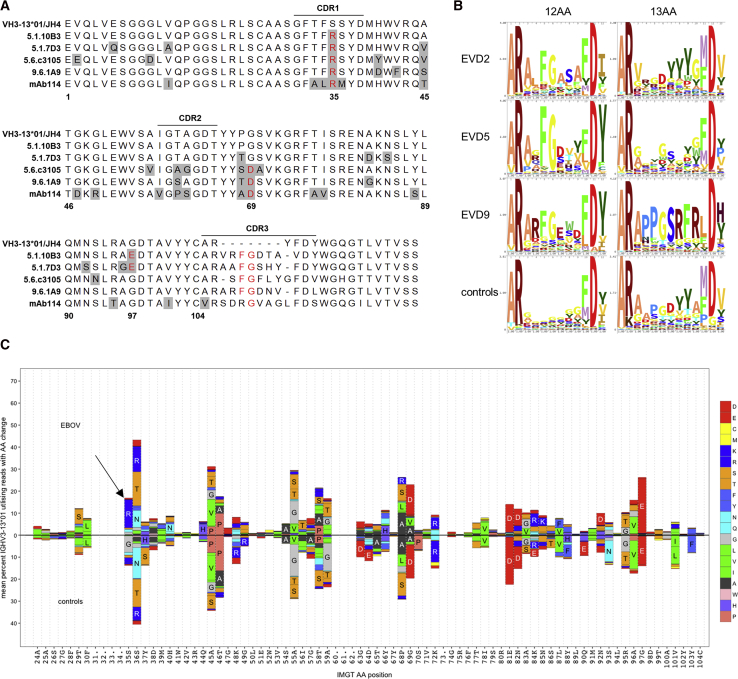
Convergent Evolution among GP1 Core-Specific mAbs (A) Sequence alignment of VH3-13 mAbs. Residues that differ from the germline are highlighted in gray. Red text indicates shared mutations or CDR3 residues. mAb114 was isolated by Corti et al. (2016) from a survivor of the 1995 Kikwit EBOV outbreak. (B) Enrichment of central FG motif in repertoire sequencing. Sequence logos illustrate the amino acid content at each position within the CDR3s of IGHV3-13^∗^01 rearrangements with 12 or 13 amino acid CDR3s. Sequences from control subjects are pooled from 7 donors. (C) Amino acid substitution profiles of IGHV3-13^∗^01 in repertoire sequencing. Sequences pooled from donors EVD2, EVD5, and EVD9 (top) are compared to sequences from controls (bottom). Germline residues and IMGT codon positions are indicated on the x axis. The arrow marks an S35R change enriched in EVD sequences. See also Figure S7 and Table S1.

We examined our antibody sequencing data for features conserved among protective antibodies. Among the 344 GP-specific antibodies, we identified only 2 small clonal families of two antibodies each. However, certain V-regions were preferentially utilized by neutralizing antibodies (Figure S7A). Three GP1 core-specific antibodies from EVD5 and one from EVD9 utilized the VH3-13 gene (Figure S7B), the same VH gene used by mAb114 (Corti et al., 2016, Misasi et al., 2016). An alignment of these heavy chains with the germline VH3-13^∗^01 sequence is shown in Figure 7A. The four mAbs from EVD5 and EVD9 share the same CDR3 length (12 amino acids) and central phenylalanine-glycine (FG) sequence motif.

We examined IGHV3-13^∗^01 sequences with 12 AA CDR3 segments in our repertoire sequencing data. In EVD2, 67.40% (488/724) of these sequences contained an FG motif at the center of CDR3. 87.66% (6,726/7,673) and 61.94% (511/825) of these sequences had this motif in EVD5 and EVD9, respectively. Across 7 control subjects there were 333 IGHV3-13^∗^01 rearrangements with 12 AA CDR3s, of which only 1 (0.30%) contained the central FG motif. Enrichment of this motif was not seen in sequences with 13 AA CDR3 lengths in EVD survivors or controls (Figure 7B). In addition to the CDR3 similarities, all the VH3-13 mAbs, including mAb114, shared a common mutation in CDR1 (S35R). This mutation was found in 12.80% ± 0.061% of all VH3-13^∗^01 sequences from EVD2, EVD5, and EVD9, compared to only 2.07% ± 0.014% of VH3-13^∗^01 sequences from controls (Figure 7C). Enrichment of this CDR1 mutation or the FG motif in CDR3 was not seen in VH3-13 sequences from EVD15, perhaps due to the lower overall EBOV-specific response or the lack of a VH3-13^∗^01 allele in this patient.

In addition to the similarities among VH3-13 mAbs, we noticed that the two antibodies in competition group C (specific for the GP1-GP2 interface) were highly similar in their heavy and light chain usage. Two non-neutralizing GP-specific mAbs (5.1.9E3 and 2.1.1B4) had highly similar sequences (Figure S7C) but bound cleaved GP only and thus were unable to neutralize the virus. Finally, we noticed significant similarities between mAb 5.6.c2618, which binds to a linear epitope in HR2 and HR2-specific mAbs isolated from another EBOV survivor (Figure S7D) (Bornholdt et al., 2016).

## Discussion

Studies in human survivors can provide valuable insights into protective immunity against viruses. Although the number of patients examined in this study was limited, we were able to track their B cell responses starting earlier and extending over longer periods than previous work. This study is one of the first to characterize the human B cell response to a primary viral infection in depth, and our key findings may hold true for other infections in humans.

EBOV infection is characterized by an initial catastrophically severe clinical course with widespread infection and seeding of virus throughout the body (Martines et al., 2015). EBOV can persist after acute infection in the central nervous system, the eye, and the testes in both humans and non-human primates (Varkey et al., 2015, Diallo et al., 2016, Zeng et al., 2017, Sissoko et al., 2017, Deen et al., 2017). The continued increase in antibody SHM, serum avidity, and neutralization titers in the first 6–12 months post-infection could have been driven either by persistent viral replication or by inert viral antigen trapped in follicular dendritic cell-associated immune complexes (Mandel et al., 1980). Persistent antigen may also explain why EBOV-specific IgG4 first appeared 1–2 years post-infection; prior studies have shown that repeated antigen exposures can lead to IgG4 class-switching (van de Veen et al., 2013, Semenova et al., 2007).

If in this work, we developed a Jurkat-GP display system that may be useful for identifying antiviral mAbs with the potential for therapeutic use. All 14 neutralizing mAbs identified in this study bound native GP on Jurkat cells. Conversely, 290 out of 311 non-neutralizing antibodies were unable to bind Jurkat-GP cells. Thus, the binding assay had a 100% sensitivity for detecting neutralizing mAbs and a 93% specificity for excluding non-neutralizing mAbs. Furthermore, Jurkat GP binding helped identify the non-neutralizing protective mAb 2.1.1B2, and can be used to identify MPER-specific mAbs whose binding may require membrane-associated epitopes that cannot be recapitulated on soluble GP proteins. This is important because our peptide scanning analysis indicated that the MPER region is a major target of the antibody response, consistent with recent findings in Bundibugyo survivors (Flyak et al., 2018).

The majority of the GP-specific mAbs were able to bind to GP only after removal of the mucin and glycan cap domains by proteolytic cleavage. Consistent with this, we also observed much higher binding of serum IgG to cleaved cell surface GP compared to native GP. This suggests that the early B cell response is directed in large part toward immunodominant epitopes on cleaved GP. It has been shown that the germline-reverted forms of broadly neutralizing antibodies bind preferentially to cleaved GP (Wec et al., 2017, Zhao et al., 2017). Identifying and removing the non-neutralizing immunodominant epitopes on GP_cl_ could improve its usefulness as an immunogen.

Interestingly, the epitopes targeted by the antibody response changed over time: during the convalescent period there was increased ASC differentiation and SHM of B cells capable of recognizing the full-length, native GP protein. A possible explanation for this is that antibodies directed against the immunodominant internal GP epitopes eventually reached saturating levels, leading to suppression of these responses by antibody feedback mechanisms (Tarlinton and Smith, 2000, Zhang et al., 2013) and allowing the B cells targeting subdominant epitopes to compete more efficiently for antigen.

The fourteen neutralizing mAbs we identified bound to previously described targets on EBOV GP, including the glycan cap, the inner chalice bowl, the fusion loop, HR2, and the MPER (Flyak et al., 2018, Zhao et al., 2017, Wilkinson et al., 2017, Corti et al., 2016, Bornholdt et al., 2016, Flyak et al., 2016, Khurana et al., 2016, Audet et al., 2014, Murin et al., 2014, Olal et al., 2012, Lee et al., 2008, Wilson et al., 2000, Maruyama et al., 1999, Wec et al., 2017). Antibodies in group C appear to bind a novel epitope at the interface between GP1 and GP2 that is shared with Sudan virus GP. One protective mAb of particular interest is 2.1.1B2, a non-neutralizing antibody whose binding site lies near the C terminus of GP1, near the epitope recognized by mouse mAbs 12B5 and 14G7 (Olal et al., 2012, Wilson et al., 2000). Several features of 2.1.1B2 are unusual: (1) it was cloned from an IgM-expressing B cell, (2) it contains minimal somatic hypermutation (zero heavy chain mutations and <1% light chain SHM), (3) it could be detected in repertoire sequencing during acute infection, and (4) it targets an epitope that has been identified by multiple groups as a site of positive evolutionary selection. Antibodies such as 2.1.1B2 may thus be part of the natural IgM repertoire and play a role in patient survival.

In this study, we identified conserved neutralizing antibody rearrangements across donors. Previous studies have demonstrated “public” or “convergent” antibodies targeting Zika virus (Robbiani et al., 2017), HIV (Scheid et al., 2011), or influenza (Sui et al., 2009). Here, we extend these findings to human antibody responses against filoviruses. The most common public clone consisted of VH3-13 antibodies specific for the inner GP chalice (group B). These mAbs are highly homologous to mAb114, which prevents lethal EVD in non-human primates when administered up to 5 days post-infection (Corti et al., 2016). The convergent VH3-13 antibodies used a variety of different light chains, suggesting that much of their specificity derives from the heavy chain, similar to what is seen with VH1-69 antibodies against the influenza hemagglutinin stalk (Sui et al., 2009). A recent study has shown that VH3-13 antibodies are elicited in non-human primates given an adenovirus vectored Ebola vaccine, suggesting this class of antibodies may even be conserved across species (Cagigi et al., 2018). However, the specific features we report in this study (CDR3 length of 12 amino acids, central FG motif, and S35R mutation in FR1) appear to be unique to the human response. Besides the VH3-13 GP1 core-specific antibodies, we identified other public antibody rearrangements encoding neutralizing antibodies, including VH3-23/VK3-20 antibodies targeting the GP1/2 interface (seen in two donors in this study) and VH1-46/VK3-11 antibodies targeting HR2 seen in this study and Bornholdt et al. (2016).

EBOV remains a public health threat, as underscored by the 2018 outbreaks in the Democratic Republic of Congo, and a vaccine that provides long-term protection against the virus would be an invaluable preventive tool. The detection of neutralizing convergent antibodies within the small number of subjects in this study may bode well for these efforts, as pathogens that elicit antibodies that are convergent, as well as protective, will likely be amenable to vaccination strategies. The frequency of convergent antibody sequence signatures prior to vaccination may be useful for predicting the vaccine response and tracking this frequency over time may provide a tool for monitoring the quality of the vaccine response. This approach could be generalizable to the humoral responses to many other pathogens and vaccination efforts in the future.

## STAR★Methods

### Key Resources Table

**Table:** 

REAGENT or RESOURCE	SOURCE	IDENTIFIER
**Antibodies**		

KZ52	Maruyama et al., 1999; IBT Bioservices	0260-001
1H3	Qiu et al., 2011; David DiLillo	N/A
c13C6	Wilson et al., 2000; David DiLillo	N/A
h13F6	Wilson et al., 2000; IBT Bioservices	0201-022
StrepMAB-Classic	IBA Life Sciences	2-1507-001; RRID:AB_513133
anti-human CD19 eFluor 450	ThermoFisher	48-0198-42; RRID:AB_11220071
anti-human IgD Brilliant Violet 510	BioLegend	348220; RRID: AB_2561945
anti-human CD38 Brilliant Violet 605	BioLegend	303532; RRID: AB_2562915
anti-human CD71 PE	BioLegend	334106; RRID: AB_2201481
anti-human CD3 Alexa 700	BioLegend	344822; RRID:AB_2563420
anti-human CD27 PerCP-Cy5.5	BD Biosciences	560612; RRID:AB_1727457
anti-human CD20 PE-Cy7	BD Biosciences	560735; RRID:AB_1727450
Goat anti-human IgG-PE	Southern Biotech	2040-09; RRID:AB_2795648
*F(ab’)*_*2*_*Goat Anti-Human IgG (Fcγ) HRP*	Jackson ImmunoResearch	109-036-098
F(ab’)_2_ Goat anti-human IgM (μ-chain) HRP	Jackson ImmunoResearch	109-036-129
F(ab’)_2_ Goat anti-human IgA (α-chain) HRP	Jackson ImmunoResearch	109-036-011
Mouse anti human IgG1 Fc-HRP	Southern Biotech	9054-05; RRID:AB_2796627
Mouse anti human IgG2 Fc-HRP	Southern Biotech	9060-05; RRID:AB_2796633
Mouse anti human IgG3 Hinge-HRP	Southern Biotech	9210-05; RRID:AB_2796699
Mouse anti human IgG4 Fc-HRP	Southern Biotech	9200-05; RRID:AB_2796691

**Bacterial and Virus Strains**		

Ebola ΔVP30-RenLuc virus	Halfmann et al., 2008	N/A
Mouse-adapted Ebola virus	Bray et al., 1999	N/A
NEB Stable cells	New England Biolabs	C3040H

**Chemicals, Peptides, and Recombinant Proteins**		

Alexa Fluor 488 Protein Labeling Kit	ThermoFisher	A10235
Alexa Fluor 647 Protein Labeling Kit	ThermoFisher	A20173
Alexa Fluor 405 NHS Ester	ThermoFisher	A30000
Live/Dead Fixable Near-IR Dead Cell Stain Kit	ThermoFisher	L10119
Lightning-Link (R) R-PE Antibody Labeling Kit	Novus Bio	703-0015
Ebola virus glycoprotein ectodomain (GP_ec_)	Fusco et al., 2015	N/A
Ebola virus delta mucin glycoprotein ectodomain (GP_Δmuc_)	Fusco et al., 2015	N/A
Ebola virus cleaved glycoprotein ectodomain (GP_cl_)	Fusco et al., 2015	N/A
Ebola virus secreted glycoprotein (sGP)	Fusco et al., 2015	N/A
Ebola matrix protein (VP40)	Bornholdt et al., 2013	N/A
NP N-terminal domain	Kirchdoerfer et al., 2015	N/A
NP C-terminal domain	Kirchdoerfer et al., 2016	N/A
VP35	Kirchdoerfer et al., 2015	N/A
VP30	Kirchdoerfer et al., 2016	N/A
Bundibugyo virus glycoprotein	IBT Bioservices	0505-015
Sudan virus glycoprotein	IBT Bioservices	0502-015
Reston virus glycoprotein	IBT Bioservices	0504-015
Ebola GP 15-mer peptide library	McElroy et al., 2015	N/A
SMCC activated cross-linked APC	Anaspec	AS-72108
MPER peptide (GP_630-649_-Cys)	Genscript	Custom synthesis
EZ-Link Sulfo-NHS-LC-LC-Biotin	ThermoFisher	A35358
Avidin D-HRP	Vector Labs	A2004
TempliPhi100 kit	GE Healthcare	25-6400-10
Thermolysin	Promega	V4001

**Critical Commercial Assays**		

BLC Human ProcartaPlex Simplex Kit	ThermoFisher	EPX01A-12147-901
Immune Repertoire Capture technology	Atreca	N/A

**Deposited Data**		

IGH repertoire sequencing data from EVD donors	This paper	Bioproject: PRJNA531326
mAb sequences	This paper	Bioproject: PRJNA531326
AbVec6W vector sequences	This paper	Bioproject: PRJNA531326
pNL-GP vector sequence	This paper	Bioproject: PRJNA531326

**Experimental Models: Cell Lines**		

Human: Expi293F	ThermoFisher	Cat # A14527; RRID:CVCL_D615
Human: Jurkat E6-1	ATCC	Cat # TIB152; RRID:CVCL_0367
Monkey: Vero	ATCC	*Cat # CRL-1586; RRID:CVCL_0574*
Monkey: Vero VP30	Halfmann et al., 2008	N/A

**Experimental Models: Organisms/Strains**		

Mouse: Female BALB/c	Charles River	Strain Code: 28

**Oligonucleotides**		

Multiplex antibody cloning primer set	Smith et al., 2009	N/A
BIOMED2 IGHV framework primers	Langerak et al., 2012	N/A
Isotype-specific primers for IgA, IgM, IgG	Roskin et al., 2015	N/A

**Recombinant DNA**		

psPax2	Addgene	RRID:Addgene_12260
pMD2.G	Addgene	RRID:Addgene_12259
pNL(CMV)EGFP/CMV/WPREΔU3	Ou et al., 2012; Addgene	RRID:Addgene_41790
pNL-sGPC	Kulkarni et al., 2014	N/A
Ebola GP (Makona strain)	Sino Biological	VG40442-UT
AbVec antibody expression vectors	Smith et al., 2009	N/A
pcDNA6.2-GW/EmGFP-miR	ThermoFisher	K493600

**Software and Algorithms**		

The PyMOL Molecular Graphics System, Version 1.8	Schrödinger	N/A
GraphPad Prism 6	GraphPad Software	N/A
IgBLAST	Ye et al., 2013	https://www.ncbi.nlm.nih.gov/igblast/
IMGT V Quest	Brochet et al., 2008	http://www.imgt.org/
FLASH	Magoč and Salzberg, 2011	http://www.cbcb.umd.edu/software/flash/
cd-hit	Fu et al., 2012	http://cd-hit.org/
Skylign	Wheeler et al., 2014	http://skylign.org/
R	R Development Core Team, 2017	https://www.R-project.org/

**Other**		

Maxisorp 96 well ELISA plates	ThermoFisher	*44-2404-21*
Clona-cell-TCS medium	Stem Cell Technologies	03814

### Contact for Reagent and Resource Sharing

Further information and requests for resources and reagents should be directed to and will be fulfilled by the Lead Contact, Rafi Ahmed (rahmed@emory.edu).

### Experimental Model and Subject Details

#### Samples from human subjects

The four EVD patients (adults; 2 male, 2 female) provided written informed consent before enrolling in the study. The study received IRB approval at Emory University (IRB00076700) and at CDC (6643.0). All PBMC and serum samples were obtained after the patients were discharged from the hospital and all work with these samples was performed under BSL2+ conditions. Prior to discharge, all four patients had tested negative qPCR for EBOV RNA in the plasma on at least two consecutive plasma samples collected at least 24 hours apart. Healthy control subjects in the antibody repertoire studies were 13 adult participants in a study of seasonal influenza vaccination. Immunoglobulin sequence data from a subset of these subjects has been previously described (Wang et al., 2014).

#### Mice

Female BALB/c mice, aged 6 to 8 weeks, were purchased from Charles River Laboratory. Upon arrival, mice were housed in microisolator cages in an animal biosafety level 4 containment area and provided chow and water *ad libitum*. Research was conducted under an IACUC approved protocol in compliance with the Animal Welfare Act, PHS Policy, and other Federal statutes and regulations relating to animals and experiments involving animals. The facility where this research was conducted is accredited by the Association for Assessment and Accreditation of Laboratory Animal Care, International and adheres to principles stated in the Guide for the Care and Use of Laboratory Animals, National Research Council, 2011.

#### Cell lines

Jurkat E6-1 cells (human acute T cell leukemia; male) were maintained in 5% CO_2_ at 37°C in RPMI-1640 medium supplemented with 100 U/ml Penicillin/Streptomycin, 2 mM L-glutamine, and 10% heat inactivated fetal calf serum (FCS). Expi293F cells (human embryonic kidney epithelial; female) were maintained on orbital shakers at 8% CO_2_ at 37°C in Expi293 medium. Vero E6 cells (green monkey kidney epithelial; female) were maintained at 5% CO_2_ at 37°C in Eagle’s minimal essential medium supplemented with L-glutamine, Penicillin/streptomycin, non-essential amino acids and 10% FCS.

### Method Details

#### Collection of human peripheral blood mononuclear cells (PBMCs):

Blood samples were collected in sodium citrate CPT cell preparation tubes (BD). 0.5 mL of blood was removed for whole blood staining and the remaining sample was immediately processed to separate PBMCs and plasma. Plasma was stored in aliquots at −80°C. PBMCs were washed four times with Dulbecco’s phosphate buffered saline without calcium or magnesium (DPBS) plus 2% FCS, suspended in 90% FCS with 10% DMSO, frozen in freezing chambers at −80°C, and transferred to liquid nitrogen.

#### EBOV-specific ELISA

100 ng of recombinant protein (GP_ec_, GP_cl_, sGP, VP40, VP35, or VP30) or 50 ng NP N-terminal domain plus 50 ng NP C-terminal domain (Fusco et al., 2015, Bornholdt et al., 2013, Kirchdoerfer et al., 2015, Kirchdoerfer et al., 2016) per well was coated on ELISA plates overnight at 4 degrees in 100 μl of DPBS (pH 7.2). For peptide ELISAs, 0.5 μg of each peptide was coated per well in 100 μl of 0.1 M sodium bicarbonate, pH 9.6. After coating, plates were blocked for two hours at room temperature with 10% FCS in PBS plus 0.05% Tween-20 (block buffer). For indirect capture assays, the capture antibody was coated overnight at 100 ng per well, followed by blocking, and incubation for two hours with 100 ng/well of GP_ec_. Plates were washed with PBS/0.05% Tween (PBST), and three-fold serial dilutions of serum or monoclonal antibodies were added to each well in 100 μl of block buffer for 90 minutes. After washing with PBST, bound antibodies were detected by incubation for 90 minutes with anti-human IgG, IgA, or IgM HRP detection antibodies diluted 1:1000 in block buffer. Biotinylated mAbs were made by labeling with NHS-LC-LC-biotin and detected using avidin-HRP diluted 1:5000 in block buffer. For detection of IgG subclass-specific responses, anti-human IgG1, IgG2, IgG3, or IgG4-HRP detection antibodies were used instead. Plates were washed with PBS/Tween, then PBS, followed by addition of o-phenylenediamine dihydrochloride substrate in phosphate-citrate buffer containing 0.01% hydrogen peroxide. Plates were allowed to develop for 7 minutes before adding 1 M HCl stop solution. The optical density (OD) of each well was read at 490 nm. ELISA titers are reported as the interpolated dilution of patient plasma where the cutoff OD value of 0.2 was reached.

#### Measurement of GP-specific IgG avidity

The concentration of plasma from each patient and time point which would give an optical density reading close to 1 was calculated from the results of the serial dilution ELISA. GP-coated plates were incubated with this plasma dilution, then washed for 5 minutes with either PBS or 8M Urea in PBS, prior to detection of bound IgG. Plates were then washed with PBS/Tween and secondary the antibody incubation and substrate addition steps were performed as usual. The avidity index was calculated for each plasma sample as the ratio of the OD on the urea wash plate divided by the OD of the same sample on the PBS wash plate. Similar results were seen when 5M Urea was used instead of 8M Urea in the wash step

#### Measurement of plasma CXCL13 levels

A commercial assay (BLC Human ProcartaPlex Simplex Kit; ThermoFisher) was used per the manufacturer’s instructions.

#### Measurement of EBOV-neutralization by patient plasma or monoclonal antibodies

Virus-specific neutralizing antibody responses were titrated essentially as previously described (Honnold et al., 2014). Briefly, plasma or antibodies were diluted serially in Minimal Essential Medium containing 5% FCS and 1X antibiotic/antimycotic (GIBCO) (MEM complete) and incubated 1 hour at 37°C with Ebola virus strain Kikwit. After incubation, the antibody-virus or plasma-virus mixture was added in duplicate to 6-well plates containing 90%–95% confluent monolayers of Vero E6 cells. Plates were incubated for 1 hour at 37°C with gentle rocking every 15 minutes. Following the incubation, wells were overlaid with 0.5% agarose in supplemented EBME media with 10% FCS and 2X Anti-Anti, and plates were incubated at 37°C, 5% CO_2_ for 7 days. On day 7, cells were stained by the addition of a second overlay prepared as above containing 4%–5% neutral red. Plates were incubated for 18-24 hours at 37°C, 5% CO_2_. The endpoint titer was determined to be the highest dilution with a 50% or greater or 80% or greater reduction (PRNT_50_, PRNT_80_) in the number of plaques observed in control wells. The assay limit of detection was calculated to be 5 plaque forming units per ml by this method.

#### Preparation of libraries and repertoire sequencing

RNA extracted from PBMCs at the peak of the B cell response was purified using TRIZOL (ThermoFisher). Infectious samples were handled under BSL-4 conditions until inactivated. For convalescent time points, RNA was extracted from PBMCs using the RNEasy Plus mini kit (QIAGEN). In the case of patient EVD9 at the discharge time point, RNA from purified antibody secreting cells (ASCs) was used instead of PBMC RNA. IGH libraries for high-throughput DNA sequencing were prepared from cDNA reverse transcribed from purified total RNA as previously described (Ellebedy et al., 2016). Briefly, cDNA was synthesized and rearranged IGH transcripts were amplified by PCR using BIOMED2 framework region 1 (FR1) IGHV forward primers (Langerak et al., 2012) and isotype-specific reverse primers for IgG, IgA and IgM located in the first constant region exon (Roskin et al., 2015). IGHV primers were multiplexed in single reactions for each isotype for each sample. Sample identity was encoded using 8-mer barcodes within the forward and reverse PCR primers. 2x300 paired-end sequencing of libraries was performed on the Illumina MiSeq instrument, using 600 cycle kits.

#### Processing of repertoire sequencing

Paired-end reads were merged using FLASH (Magoč and Salzberg, 2011) and samples were de-multiplexed based on 100%, full-length matches to the forward and reverse 8-mer barcodes. Primers were identified and trimmed allowing for a single mismatch within the primer (sequences without identifiable primers were discarded). Isotype subclasses were determined based on 100%, full length matches to the CH1 exon sequence upstream of primers (sequences without confirmed subclasses were discarded). Primer trimmed sequences were aligned against the human germline V, D, and J reference repertoires from IMGT/GENE-DB (Giudicelli et al., 2005) using a local installation of IgBLAST (Ye et al., 2013). IgBLAST output was used to determine germline gene utilization, to delimit complementarity determining regions (CDRs) and FR regions (IMGT definitions), define mutational changes and to determine rearrangement productivity. Non-productive (VDJs harboring stop codons or with out-of-frame IGHJ gene segments) and artificial or artifactual PCR amplicons were excluded from analysis. Artificial amplicons were identified based on the pattern of CDR3 nucleotide sequence association across reads utilizing disparate IGHVs.

Clonal relationships between rearranged genes in the repertoire sequencing were inferred using single linkage clustering. For each subject, all IGH that utilized the same IGHV and IGHJ and which had the same CDR3 lengths were clustered based on their CDR3 nucleotide sequence at a 90% identity threshold.

#### Inference of mAb lineages in repertoire sequencing

The membership of an mAb IGH to a clonal lineage from the repertoire sequencing was inferred using cd-hit (Fu et al., 2012) clustering of the CDR3 nucleotide sequences from the mAb to all IGH combined across the subjects that utilized the same IGHV, IGHJ and had same CDR3 length. Clustering was nucleotide sequence based at a 90% identity threshold, without gaps. Unlike clonal lineage inferences, the clustering of the mAbs was not restricted to within a subject’s individual repertoire but was extended across all subjects to test for potential convergent responses that may have arisen independently in the different subjects.

#### Repertoire sequencing data analysis

Analysis was performed and visualized in R (R Development Core Team, 2017) using R base packages for statistical analysis and the ggplot2 package for visualization (Wickham and Chang, 2008). Sequence logos were produced using Skylign (Wheeler et al., 2014) and multiple sequence alignment was performed using Clustal W version 2 (Larkin et al., 2007).

#### Analysis of ASC, ABC, and GP-binding B cell numbers:

For samples analyzed during the acute phase, ABC and ASC numbers were measured as described in a BSL-4 laboratory using an Accuri cytometer (McElroy et al., 2015). For convalescent time points, numbers of ASCs and ABCs were measured in whole blood from fresh samples. ASCs were gated as singlet lymphocytes that were CD3, CD19+, IgD, CD20, CD38^hi^, CD27^hi^ singlets. ABCs were gated as singlet lymphocytes that were CD3, CD19+, IgD, CD20+, CD71+ singlets. Erythrocytes were removed by treatment BD FACS lyse prior to running samples on a BD LSR II instrument. For staining GP-specific cells, GP_cl_ and GP_ec_ were labeled with Alexa Fluor 488 and Alexa Fluor 647, respectively, using amine-reactive protein labeling kits. The gate used for GP-binding cells was CD3, CD19+, IgD, CD20+, GP_cl_+ live singlets.

#### Cell sorting for mAb cloning

PBMC samples from the following time points were sorted at Emory University for mAb cloning: EVD2 1 month, 6 months, and 10 months (MPER sort); EVD5 1 month and 6 months; EVD9 6 months. The following B cell subsets were single cell sorted at Emory for antibody cloning: i) GP-binding B cells (gate: live, singlets, CD3, CD20+, CD19+, IgD, GP_cl_+ or GP_ec_+). ii) GP-negative activated B cells (gate: live, singlets, CD3, CD20+, CD19+, IgD, GP_cl_ and GP_ec_, CD71+). iii) total ASCs (gate: live, singlets, CD3, CD20, CD19+, CD38^hi^, CD71+, IgD). GP-binding B cells were sorted from all time points; GP-negative ABCs were sorted from EVD5 1 month; total ASCs were sorted from EVD2 1 month and EVD5 1 month. Cells were sorted into lysis buffer in 96-well plates for cloning of antibody heavy and light chain genes as described (Smith et al., 2009). For sorting MPER-peptide specific B cells a peptide spanning EBOV GP residues 630-649 was synthesized with a C-terminal cysteine and conjugated to APC using a commercial kit. MPER-binding B cells were sorted from the 10 month sample from EVD2 using this probe.

PBMC samples from the following time points were sorted at Atreca for next generation sequencing of paired heavy and light chain sequences: EVD5 6 months, EVD9 1 month, EVD15 discharge. Cell sorts at Atreca were performed as at Emory except that only GP_cl_ was used as the sort antigen. Bulk-sorted GP-binding B cells were cultured for 4 days in IMDM medium (Invitrogen) in the presence of FBS, Normocin, IL-2 (PeproTech), IL-21 (PeproTech), rCD40 ligand (R&D Systems), and His-Tag antibodies (R&D Systems) prior to single cell sorting. Paired chain antibody sequencing was carried out on cells sorted into 384-well plates at one cell per well by applying Immune Repertoire Capture technology as previously described (DeFalco et al., 2018).

#### Cloning of antibody heavy and light chain genes by PCR

For sorts done at Emory, mAb heavy and light chain genes were amplified by multiplex RT-PCR from individual sorted B cells or plasmablasts and cloned into human Igγ1, human Igκ, or human Igλ expression vectors as described (Smith et al., 2009), with minor modifications as follows: i) to amplify antibodies from IgA and IgM expressing cells, separate nested PCRs were run using constant region primers for IgM (Smith et al., 2009) and IgA (Benckert et al., 2011) instead of IgG. ii) an M13 Reverse primer (CAGGAAACAGCTATGACC) was added to the 5′ terminus of all 5′ primers used in the nested PCR in order non-specifically increase primer annealing to the template and increase the PCR efficiency (Regier and Shi, 2005). iii) to increase miniprep plasmid yields and reduce satellite colonies after transformations, the AgeI-HindIII fragment of vectors Abvec-hIgG1, AbVec-hIgKappa, and AbVec-hIgLambda containing the promoter and ORF regions were subcloned into the vector backbone of pcDNA6.2-GW/EmGFP-miR (ThermoFisher), which contains a pUC origin of replication and spectinomycin resistance. A WPRE element was cloned from vector pNL(CMV)EGFP/CMV/WPREΔU3 (Ou et al., 2012), a gift from Jakob Reiser, and placed downstream of the antibody ORF region for increased antibody yield in transfections. The new vectors with the pcDNA6.2 backbone and WPRE elements are referred to as AbVec6W vectors.

#### Cloning from paired heavy and light chain sequences

A total of 486 unique antibody sequences were identified by next generation sequencing, including 425 from EVD5 day 263, 30 from EVD9 day 71, and 31 from EVD15 day 14. Antibody heavy and light chains were initially synthesized for 10 mAbs chosen at random from each donor/time point to confirm that the majority of mAbs bound to GP. An additional 10 randomly selected mAbs were produced from EVD15 in order to ensure sampling of mAbs across the wide range of SHM levels observed in this donor. An additional 90 mAbs were produced from EVD5 because the initial set of 10 yielded one therapeutically-promising antibody. In total, we produced antibody for 100 out of 425 mAb sequences from EVD5 (24%), 10 out of 30 from EVD9 (33%), and 20 out of 31 sequences from EVD15 (65%). Codon-optimized DNA sequences encoding the heavy and light chain variable regions (FR1 through FR4) were synthesized with flanking restriction enzyme sites for subcloning. Codon optimization and DNA synthesis were performed by Genscript and Gen9. The synthesized heavy chain variable region genes were subcloned into AbVec6W-hIgG1 vectors and light chain genes were cloned into AbVec6W-hkappa or AbVec6W-hlambda.

#### Note on antibody nomenclature:

Antibody names contain the patient ID, the time point (in months after hospital discharge) when the PBMC sample was obtained that was used for B cell sorting, and the clone ID. For example, mAb 2.1.1B2 is from EVD2, from a B cell sorted from 1 month post-discharge, and was cloned from sort plate 1, well B2. mAbs sequenced at Atreca have clone IDs beginning with “c” (e.g., 5.1.c2618).

#### Analysis of monoclonal antibody sequences

For antibodies cloned by multiplex PCR directly from cells, the nested PCR sequence was analyzed to determine the original heavy chain constant region and IgG or IgA subclass, if applicable. mAb variable region sequences were determined from the consensus expression clones in vector AbVec6W. Portions of the sequence which were derived from PCR primers were removed before analysis. Antibody V(D)J usage and somatic hypermutation were analyzed using IMGT V-quest (Brochet et al., 2008). For antibodies cloned by next generation sequencing at Atreca, V(D)J assignment and mutation identification was performed using an implementation of SoDA (Volpe et al., 2006). IgG subclass was determined by top-scoring BLAST alignment to constant region nucleotide sequence (Camacho et al., 2009).

#### Screening neutralization assay using biologically contained EBOV

To identify neutralizing antibodies, a biologically contained EBOV, EbolaΔVP30 virus, containing the Renilla luciferase reporter gene (RenLuc) instead of the VP30 gene and expressing the Makona glycoprotein (GP; H.sapiens-tc/GIN/2014/Gueckedou-C07) instead of the Mayinga GP, was used as previously described (Halfmann et al., 2008). Briefly, in a 96-well format, EbolaΔVP30-RenLuc virus (10,000 focus-forming units) was incubated with 10 μg/mL of monoclonal antibody (mAb) for 2 hours at 37°C. The virus/mAb mixture was then inoculated onto Vero cells that stably express VP30 (VeroVP30 cells) to facilitate EbolaΔVP30-RenLuc virus replication. Three days later, luciferase expression, given as relative light units (a readout for virus infection and replication), was measured using live cell luciferase substrate, EnduRen (Promega), on a Tecan M1000 Pro plate reader. A neutralizing monoclonal antibody was determined as positive hit if the RLU was reduced by 90% compared to the negative control (EBOV VP35-specific mAb 5-69.3.2) (Takada et al., 2007). No antibodies in this study exhibited partial neutralization, which we consider to be a > 50% reduction in luciferase activity without reaching the 90% threshold.

#### Identification of viral escape mutants

The generation of antibody escape mutant viruses was previously described (Halfmann et al., 2008). Up to 15 individual plaque-picked escape mutant viruses were isolated and the GP was sequenced.

#### Monoclonal antibody production

The Expi293 expression system was used for antibody production according to the manufacturer’s instructions. A 2:1 ratio of light chain to heavy chain plasmid was used in transfections. mAb-containing supernatants from transfected expi293 cells were clarified by centrifugation and then incubated with protein A agarose resin (Genscript) in batch format overnight, followed by washing, elution, and buffer exchange into DPBS as described (Smith et al., 2009). Antibodies used *in vivo* were verified endotoxin-free using a commercial kit (ThermoFisher). Note that all antibodies produced in this study were expressed as human IgG1.

#### Generation of Jurkat cell lines stably expressing EBOV GP

A plasmid encoding EBOV GP (Makona strain) was obtained from Sino Biological. The GP gene was subcloned into cloned into previously described lentivirus vector pNL-sGPC (Kulkarni et al., 2014). The resulting plasmid is termed pNL-GP. Lentiviral particles were produced by transfection of expi293 cells with pNL-GP and packaging plasmids pMD2.G and psPax2 (gifts from Didier Trono). The viral stock was concentrated using PEG-it virus precipitation solution (System Biosciences). Jurkat cells were transduced at a multiplicity of infection of 10, and a stable expressing clone (Jurkat-GP) was obtained after three rounds of single cell sorting (FACSAria) into 96 well plates. Alexa 488 labeled mAb 5.6.1A2 (this study) was used to sort high expressing cells. 50% Jurkat-conditioned medium was added to wells to promote cell outgrowth after single cell sorting.

#### Cell-based antibody binding assays

For testing mAb binding to native GP, Jurkat-GP cells were incubated with mAbs at 4°C at a concentration of 5 ug/ml in DPBS supplemented with 2% fetal calf serum and 2 mM EDTA (FACS buffer). For testing plasma IgG binding, cells were uncubated with at 1:5000 dilution of plasma in FACS buffer. Cells were washed in FACS buffer and incubated with anti-human IgG-PE, washed again, and analyzed on a FACSCanto instrument.

For competition binding assays, unlabeled competitor antibody was preincubated with Jurkat-GP cells for 30 minutes at 100 ug/ml. Incubations were done on ice in FACS buffer containing 0.05% sodium azide to prevent GP internalization or shedding. Alexa 488 labeled test antibody was then added to a final concentration of 2 ug/ml without removing the competitor mAb. After 30 minutes, cells were washed and FITC channel fluorescence was assessed on a BD FACSCanto. The specific fluorescence in each condition was calculated by subtracting the geometric mean fluorescence in the FITC channel of cells incubated without Alexa 488 labeled antibody. The percentage of binding in the presence of each competitor was calculated as the specific FITC fluorescence in the presence of the competitor, divided by the specific fluorescence without competitor, times 100%.

#### Treatment of cells with thermolysin or dithiothreitol (DTT):

In some experiments, parental Jurkat E6-1 cells or Jurkat-GP cells were treated with thermolysin to remove the glycan cap and mucin domains of GP (Kaletsky et al., 2007). Cells were washed in DPBS containing calcium and magnesium (DPBS++), then resuspended at one million cells per ml in DPBS++ containing 0.5 mg/ml thermolysin. Cells were incubated 30 minutes at 37°C, then the reaction was stopped by washing with FACS buffer prior to staining.

In other experiments, cells were treated with the reducing agent DTT (Promega), which has been shown to cause partial release of GP1 from the cell surface and increase exposure of epitopes on the GP base (Francica et al., 2010). Parental Jurkat cells or Jurkat-GP cells were washed in FACS buffer, incubated for 30 minutes in FACS buffer containing 1 mM DTT at 37°C, then washed in FACS buffer prior to staining.

#### Jurkat cell display of EBOV-GP mutants

Error prone rolling circle amplification was used to mutagenize the pNL-GP using the TempliPhi kit and random hexamers (Fujii et al., 2006). An extra 0.5 mM of dNTPs was added to the reaction to enhance the amplification efficiency.(Rector et al., 2004) The amplified product was digested with BamHI to separate individual plasmid copies and then self-ligated. NEB Stable cells were transformed with the ligated plasmid. A plasmid library was generated from 80,000 clones and used to generate lentiviral particles with pMD2.G and psPax2 as described above. The lentivirus stock was to transduce Jurkat cells at a multiplicity of infection of 0.05. Five to ten days post-transduction, Jurkat cells expressing GP mutants which lacked binding to a specific mAb but retained binding to a panel of 2-3 control mAbs from separate competition groups were enriched by multi-color sorting with mAbs directly labeled with Alexa-405, −488, −647, or PE. Sorted cells were then single cell cloned using Clona-cell-TCS medium. Individual Jurkat-GP mutant clones were rescreened to confirm that they lacked binding to the mAb of interest and maintained binding to the panel of control mAbs. RNA was extracted from cell lines and amplified with primers flanking the GP insert to determine the specific amino acid changes present in GP. Cell lines expressing GP molecules with multiple amino acid changes were excluded from analysis.

For determining mAb binding to GP mutants, Jurkat cells expressing wild-type or mutant GPs were incubated with unlabeled mAbs at 5 ug/ml, followed by staining with anti-human IgG-PE and detection of fluorescence by flow cytometry as above. The binding of a control mAb (2.1.6F2 or KZ52) was used to control for variable GP expression across cell lines. An irrelevant human IgG1 was used as a negative control to assess background binding. The percentage binding of a test mAb to a mutant GP was defined as: 100% × [(binding of test mAb to mutant) ÷ (binding of test mAb to wild-type GP)] × [[(binding of control mAb to mutant) ÷ (binding of control mAb to wild-type GP)].

#### Visualization of GP

Pymol software version 1.8 was used to visualize mutations on the Ebola GP structure (PDB ID 5JQ3) (Zhao et al., 2016).

#### Testing of mAbs for protection of mice from EBOV infection

The lethal mouse-adapted EBOV mouse model was developed at the U.S. Army Medical Research Institute of Infectious Diseases (USAMRIID) by serial passages of EBOV (Zaire) in progressively older suckling mice (Bray et al., 1999). On day 0, mice were infected intraperitoneally (i.p.) with 100 p.f.u. of mouse-adapted EBOV. Twenty-four hours prior to infection, groups of mice (10 mice per group) were treated i.p. with a single dose of antibody. Negative control mice received irrelevant control human IgG1 antibody. Mice were monitored daily (twice daily if there were clinical signs of disease) for 21 or 28 days post-infection. Mice were weighed as a group. The average mouse weight in grams was calculated by dividing the group weight by the number of mice surviving mice in a treatment group at each time point. Disease scores were recorded based on the sickest animal in each treatment group (normal (0), reduced grooming/ruffled fur (1), subdued, but normal when stimulated (2), lethargic: hunched posture; subdued even when stimulated (3), nasal discharge/bleeding/unresponsive, when stimulated/weak/paralysis (4)). A half score (1.5, 2.5 etc.) means that sickest animal was between scoring parameters.

### Quantification and Statistical Analysis

Two group comparisons were conducted using unpaired t test and Wilcoxon Rank Sum test. Log-rank test was used to compare survival curves. GraphPad Prism software and R were used for performing statistical tests.

### Data and Software Availability

Repertoire sequencing data, mAb sequences, and plasmid sequences have been deposited in SRA and Genbank (Bioproject: PRJNA531326).

## Supplemental Information

Table S1. mAb Sequences and Screening Data, Related to Figures 3, 4, 5, 6, and 7 Excel file containing sequences of monoclonal antibodies, analysis of somatic hypermutation, original antibody isotypes, and results of screening neutralization, ELISA, and cell surface binding assays.
